# Quality Improvement Intervention to Increase Sleep Apnea Diagnostic Testing After Stroke and Transient Ischemic Attack: A Cluster Randomized Trial

**DOI:** 10.1001/jamanetworkopen.2025.43385

**Published:** 2025-11-14

**Authors:** Dawn M. Bravata, Anthony J. Perkins, Laura J. Myers, Joanne K. Daggy, Ali Sexson, Stanley E. Taylor, Laura Burrone, Brian B. Koo, Edward J. Miech, Nick Rattray, K. Maya Story, Kimberly J. Waddell, Qinglan Priscilla Ding, Jason J. Sico

**Affiliations:** 1 Department of Veterans Affairs (VA) Health Services Research and Development (HSR&D) Expanding Expertise Through E-health Network Development Quality Enhancement Research Initiative, Indianapolis, Indiana; 2 VA HSR&D Center for Health Information and Communication, Richard L. Roudebush VA Medical Center, Indianapolis, Indiana; 3 Regenstrief Institute, Indianapolis, Indiana; 4 Department of Medicine, Indiana University School of Medicine, Indianapolis; 5 Medicine Service, Richard L. Roudebush VA Medical Center, Indianapolis, Indiana; 6 Department of Neurology, Indiana University School of Medicine, Indianapolis; 7 Department of Biostatistics and Health Data Science, Indiana University School of Medicine, Indianapolis; 8 Neurology Service, VA Connecticut Healthcare System, West Haven, Connecticut; 9 Pain Research, Informatics, and Multi-morbidities, and Education Center, VA Connecticut Healthcare System, West Haven, Connecticut; 10 Department of Neurology, Yale School of Medicine, New Haven, Connecticut; 11 VA Center for Health Equity Research and Promotion, Crescenz VA Medical Center, Philadelphia, Pennsylvania; 12 Department of Physical Medicine and Rehabilitation, Perelman School of Medicine, University of Pennsylvania, Philadelphia; 13 Leonard Davis Institute for Health Economics, University of Pennsylvania, Philadelphia; 14 Purdue University School of Nursing, West Lafayette, Indiana; 15 Department of Internal Medicine, Yale School of Medicine, New Haven, Connecticut

## Abstract

**Importance:**

Obstructive sleep apnea (OSA) is common among patients with ischemic stroke and transient ischemic attack (TIA) and is associated with poor outcomes. Guidelines favor considering sleep testing among patients with cerebrovascular events.

**Objective:**

To evaluate the effectiveness of a quality improvement intervention to increase OSA diagnostic testing after stroke or TIA.

**Design, Setting, and Participants:**

This stepped-wedge cluster randomized trial of patients with ischemic stroke or TIA admitted to Department of Veterans Affairs facilities assessed outcomes during 8 data periods of 7-month duration (May 15, 2019, to January 24, 2024).

**Intervention:**

The quality improvement intervention was implemented with a virtual kickoff; site teams reviewed their facility’s baseline data, identified improvement opportunities, considered barriers and potential solutions to diagnosing OSA among patients with cerebrovascular disease, and developed a site-specific action plan. Team members attended monthly collaborative conferences. A web-based platform provided quality data, action plans, and a resource library. The research team provided external facilitation.

**Main Outcomes and Measures:**

The primary outcome was 30-day OSA diagnostic testing rate. Secondary outcomes were 30-day positive airway pressure treatment rate, 90-day recurrent vascular event rate, and 90-day readmission rate. Implementation effects were assessed using generalized linear mixed-effects models with binomial distributions and log link fit to patient-level data with site-level random effects.

**Results:**

The study included 1747 patients at 6 intervention sites (mean [SD] age, 68.7 [11.1] years; 1634 [93.5%] male) and 7454 patients at 30 usual care sites (mean [SD] age, 71.8 [10.8] years; 7114 [95.4%] male). The index event was stroke (vs TIA) in 1429 (81.8%) at the intervention sites and 5931 (79.6%) at the usual care sites. The 30-day diagnostic testing rate increased from 2.1% (20 of 952) during baseline to 29.1% (189 of 650) during implementation (odds ratio [OR], 16.13; 95% CI, 8.25-31.53); the 30-day diagnostic testing rate varied from 0.7% to 2.2% among usual care sites across data periods. Thirty-day positive airway pressure treatment increased from 0.3% (3 of 952) during baseline to 2.8% (18 of 650) during implementation (OR, 14.22; 95% CI, 2.40-84.40) at the intervention sites, while varying from 0.0% (0 of 876) to 0.4% (4 of 1158) at the usual care sites. No statistically significant changes were observed in 90-day readmission or recurrent vascular event rates.

**Conclusions and Relevance:**

In this cluster randomized trial of patients with acute cerebrovascular events, quality improvement approaches increased OSA testing. These results suggest that health care systems can improve the delivery of guideline-concordant care for patients with acute ischemic stroke and TIA by delivering inpatient sleep medicine services.

**Trial Registration:**

ClinicalTrials.gov Identifier: NCT04322162

## Key Points

**Question:**

Can a quality improvement intervention improve sleep apnea diagnostic testing among patients who are hospitalized for acute ischemic stroke or transient ischemic attack?

**Findings:**

In this stepped-wedge cluster randomized trial with 1747 patients at 6 intervention sites and 7454 patients at 30 usual care sites, the 30-day diagnostic testing rate increased from 2.1% (baseline) to 29.1% (21-month active implementation), whereas usual care sites maintained a low rate.

**Meaning:**

These results suggest that sites seeking to implement acute care sleep services can use quality improvement strategies to provide sleep apnea diagnostic testing to patients with acute cerebrovascular disease.

## Teaser

This cluster randomized trial of patients with ischemic stroke or transient ischemic attacks admitted to Department of Veterans Affairs facilities examines the effectiveness of a quality improvement intervention to increase obstructive sleep apnea diagnostic testing.

## Introduction

Approximately 70% of patients with ischemic stroke and transient ischemic attack (TIA) have obstructive sleep apnea (OSA).^1,2^ However, most patients with cerebrovascular disease with OSA are neither diagnosed nor treated.^3,4,5^ Untreated OSA has been associated with poor outcomes among patients with cerebrovascular disease, including higher mortality and worse functional status.^6,7,8,9,10^ Continuous positive airway pressure (CPAP) among patients with ischemic stroke or TIA and OSA improves outcomes, including neurologic symptom severity and mortality.^11,12,13,14,15,16,17,18,19,20,21,22^

These data provided the rationale for guideline recommendations to consider diagnostic OSA testing among patients with cerebrovascular events and treating OSA with CPAP.^23,24^ Vast literature supports using CPAP to improve OSA outcomes.^25^ Evidence suggests that CPAP used for stroke-related outcomes (eg, neurologic recovery) is most likely to be effective if begun soon after the cerebrovascular event.^4,14,16,26,27^ However, substantial implementation barriers hinder acute diagnostic sleep testing and early treatment after cerebrovascular events because most sleep medicine departments operate in the outpatient setting and wait times delay diagnosis and treatment.^28,29,30,31^

Our primary objective was to examine the effectiveness of a quality improvement (QI) intervention to increase OSA diagnostic testing among patients with acute ischemic stroke or TIA. Our secondary objectives were to examine sustainability of the QI intervention and explore intervention effects on OSA treatment, recurrent vascular events, and readmissions.

## Methods

### Stepped-Wedge Cluster Randomized Design Augmented With Usual Care Sites

**Figure 1.**
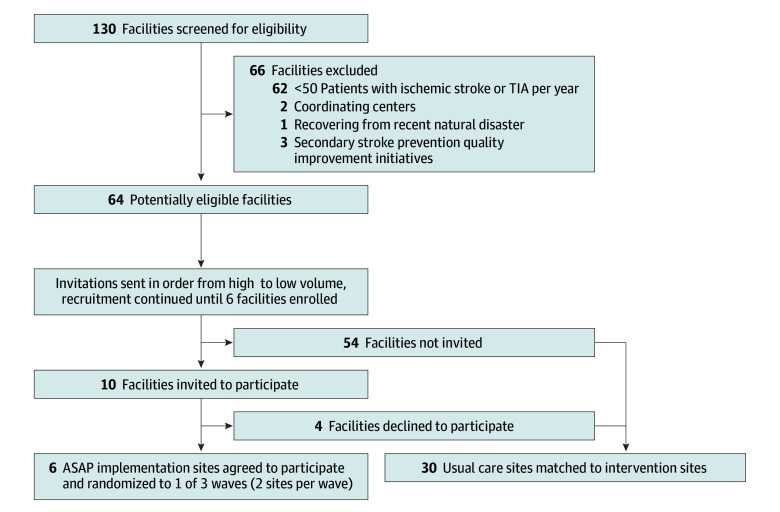
Flow Diagram for the Addressing Sleep Apnea Post-Stroke and Transient Ischemic Attack (ASAP) Trial The flow diagram displays the information about site recruitment and an overview of the stepped-wedge cluster-randomized trial design with 6 intervention sites (2 sites per wave) and 30 usual care sites.^32^ Each of the 8 data periods lasted 7 months. No sites withdrew from the study. TIA indicates transient ischemic attack.

The Addressing Sleep Apnea Post-Stroke and Transient Ischemic Attack (ASAP) stepped-wedge cluster randomized trial evaluated a QI intervention to increase OSA diagnostic testing among patients with stroke or TIA admitted to 6 Department of Veterans Affairs (VA) hospitals and 30 VA usual care sites. Recruitment occurred at the facility level, with a target of 6 intervention sites (Figure 1). Invitation letters were sent to 10 facilities with highest stroke or TIA volumes; 6 agreed to participate. A randomization list determined the order sites started the intervention and hence assignment to 1 of 3 waves (2 sites per wave). Usual care and intervention sites used the same electronic health record (EHR) and had identical facility complexity (based on 8 characteristics [eg, teaching status and intensive care unit level] with complexity levels of 1a [most complex], 1b, 1c, 2, or 3 [least complex]); 5 intervention sites were 1a, and 1 was 1b. All intervention sites had neurologic services (half were admitting and half were consultative; half had a stroke coordinator) and sleep medicine services (none provided inpatient polysomnography). Five usual care sites were matched to each intervention site on annual stroke or TIA patient volume and baseline OSA diagnostic testing rates. There was no patient or public involvement in the design, conduct, or reporting of this research. The ASAP trial was approved by the VA Central Institutional Review Board. Patients were not asked to give informed consent for participation because the QI intervention was implemented facility-wide. This study follows the Consolidated Standards of Reporting Trials (CONSORT) reporting guideline.^32^ The trial protocol can be found in Supplement 1.

The stepped-wedge design provided eight 7-month data periods (May 15, 2019, to January 24, 2024) (Figure 1) with 3 phases at intervention sites. Baseline varied in duration, extending from May 15, 2019, to sites’ kickoffs (first site: February 19, 2021; last site: April 29, 2022). The 21-month active implementation phase began with the kickoff. Sustainability was truncated to one data period after active implementation at only 4 sites due to design modifications, approved by the VA Health Systems Research Data and Safety Monitoring Board, in response to COVID-19 pandemic^33^ disruptions (beginning March 2020) and the Philips Respironics international CPAP device recall (beginning June 2021).^34^

### Outcomes

The primary effectiveness outcome was the 30-day OSA diagnostic testing rate, which was defined as receipt of any sleep study (attended or unattended, in a laboratory or at home) within 30 days of admission for the index cerebrovascular event. The sleep study had to be completed (not just ordered or scheduled). Secondary effectiveness outcomes were 30-day CPAP treatment rate (CPAP initiation within 30 days of admission; treatment could have been provided during the inpatient period or after discharge), 90-day recurrent vascular event rate (emergency department or hospital admission for TIA, stroke, myocardial infarction, acute coronary syndrome, arrythmia, congestive heart failure, or all-cause mortality within 90 days of discharge), and 90-day all-cause unplanned readmission rate. Exploratory outcomes were 90- and 180-day OSA diagnostic testing rates and 90- and 180-day CPAP treatment rates.

### QI Intervention

The QI intervention involved diverse clinical staff at intervention sites participating in a virtual kickoff based on systems redesign principles^35^ used in VA QI projects.^36,37,38,39^ During kickoffs, sites’ team members reviewed their facility’s baseline data, identified improvement opportunities, considered barriers to identifying patients, diagnosing OSA among cerebrovascular disease, brainstormed solutions to barriers, ranked solutions on impact-effort matrices, and developed site-specific action plans focusing on high-impact and low-effort activities in the short term and high-impact and high-effort activities in the long term.^40,41,42,43^ Site-specific action plans were designed by site staff to address site-specific contextual characteristics. For example, at one site, a stroke nurse was notified of patients with cerebrovascular disease; however, other sites had to implement an EHR tool to identify potentially eligible patients. Team members joined monthly collaborative conferences to share experiences (accomplishments and barriers), identify next month’s goals, review emerging evidence or tools, and brainstorm solutions for shared difficulties.^43^ A web-based platform provided site-specific data, action plans, and resources (eg, educational materials, guidelines, and EHR tools).^40^ The research team provided external facilitation at least monthly.

### Sample Size

The ASAP trial was designed based on the following estimates: 64 patients with stroke or TIA would be admitted per site per data period, and the mean facility 30-day diagnostic testing rate would be 70% for eligible patients during active implementation vs 7% for baseline. Six intervention sites provided greater than 90% power to detect a difference in the 30-day diagnostic testing rate as small as between 7% (baseline) and 15% (active implementation), while varying the coefficient of variation (or intracluster correlation). Power was conservative and based on a 2-sided Wald *z* test of the parameter corresponding to the indicator of implementation (reference: baseline) among intervention sites. Power was calculated using R software, version 4.3.1 (R Foundation for Statistical Computing).^44^ Therefore, the ASAP trial was powered to detect clinically meaningful changes in the primary outcome for the primary analysis (30-day OSA diagnostic testing rate: baseline vs active implementation), but power was limited for secondary outcomes (eg, CPAP treatment) and secondary analysis (baseline vs sustainability).

### Data Sources

The primary data source was the VA Corporate Data Warehouse, a national repository of clinical and administrative information.^45^ Care Assessment Needs (CAN) scores (validated measures of mortality risk) were also obtained from the Corporate Data Warehouse.^46^ The National Institutes of Health Stroke Scale (NIHSS),^47^ a measure of stroke symptom severity, was obtained by text mining clinical notes. Mortality status was obtained from the VA Death Ascertainment File,^48^ which includes Master Person Index and Social Security Administration Death Master File data.^49^ Results of sleep studies conducted at intervention sites during active implementation were collected via EHR review.

### Patient Cohort

Patients with a primary diagnosis code for ischemic stroke or TIA in the inpatient setting were identified. Data on self-identified (in 99.7% of patients) race and ethnicity were collected to further describe the cohort. Exclusions were hospitalization for less than 24 hours; transferred to a non-VA acute care facility within 2 days of admission; in-hospital death; left against medical advice (if a patient returned ≤24 hours, then admissions were linked and included); COVID-19 positive test result in the 30 days before, during, or after discharge; subarachnoid hemorrhage during admission; carotid endarterectomy or stenting on day of admission; or hospice or palliative care in 1 year before admission, during hospital stay, or at discharge. Patients contributed one observation to analyses. For patients with multiple admissions, events were randomly selected within the following hierarchy: (1) active implementation events, (2) sustainability events, and (3) baseline events. We identified patients eligible for 30-day OSA diagnostic testing by excluding patients with a history of OSA (based on diagnosis code; uvulopalatopharyngoplasty, hypoglossal nerve stimulator, oral mandibular device, or CPAP in past 5 years), life-sustaining treatment orders for comfort care only, or death within 7 days after discharge.

### Statistical Analysis

The main comparison was the mean facility 30-day OSA diagnostic testing rate at 6 intervention sites during baseline vs active implementation data periods. We verified that patient characteristics were similar at usual care and intervention sites using appropriate statistical models based on data type (linear, logistic, or generalized logit), including a random facility effect to adjust for correlation of patients within facilities. A similar analysis was conducted to compare patients across study phases (baseline, active implementation, and sustainability) within intervention sites and to compare consistency of cohorts across data periods for usual care sites.

A generalized linear mixed-effects model with a binomial distribution and logit link fit to patient-level data with site-level random effects was used to evaluate the effect of active implementation on 30-day diagnostic testing rates. The model included time (data period) as a categorical variable (reference: period 1); indicator for active implementation (reference: baseline), which was the primary effect of interest; indicator for sustainability (reference: baseline); a site-level indicator for implementation vs usual care site; and a random intercept for site to account for correlation of patient outcomes within sites. Model fit was used to select whether the random effect would be allowed to vary by site type. Similar models were constructed for secondary outcomes. Statistical significance was defined as a 2-sided *P* < .05. We constructed individual risk adjustment models for each primary and secondary outcome (excluding treatment rate given the expected low rate) using complete case data from baseline for all 36 sites. Risk-adjusted variables for each outcome were included in models to examine whether results were similar after including potential confounders. Two patient covariates had missingness (NIHSS score: 26.0%; CAN score: 7.5%); therefore, multiple imputation with 20 imputations was conducted using fully conditional specification for adjusted models (eAppendix in Supplement 2).^50^ Because treatment data were expected to be sparse, the exact Wilcoxon signed-rank test was used to compare treatment rates between baseline and active implementation at intervention sites.

We preplanned 3 sensitivity analyses. First, to examine potential effects of the COVID-19 pandemic, we included a time-varying measure of COVID-19 burden (number of patients with COVID-19 per data period per site). Second, we examined potential effects of the international Philips Respironics device recall^34^ by excluding data period 5 and including a categorical variable indicating level of access to VA CPAP machines for each data period (during red periods only patients at imminent risk of adverse events if OSA was untreated had access to CPAP devices, during yellow periods patients with stroke received priority CPAP access, and during green periods CPAP machines were widely available). Third, given that action plan implementation required time after kickoff, we excluded the first active implementation data period and compared baseline to the second 2 active implementation data periods.

## Results

The study included 1747 patients at 6 intervention sites (mean [SD] age, 68.7 [11.1] years; 1634 [93.5%] male and 113 [6.5%] female; 744 [42.6%] Black, 880 [50.4%] White, 30 [1.7%] other [American Indian and Alaska Native, Asian, and Native Hawaiian and Other Pacific Islander], and 93 [5.3%] unknown) and 7454 patients at 30 usual care sites (mean [SD] age, 71.8 [10.8] years; 7114 [95.4%] male and 340 [4.6%] female; 2243 [30.1%] Black, 4725 [63.4%] White, 187 [2.5%] other [American Indian and Alaska Native, Asian, and Native Hawaiian and Other Pacific Islander], and 299 [4.0%] unknown)^51^ (Table 1). The index event was stroke (vs TIA) in 1429 (81.8%) in the intervention group and 5931 (79.6%) in the usual care group. Reasons for ineligibility for diagnostic testing (not mutually exclusive) were OSA history in 3392 (30.2%) in usual care sites and 761 (28.8%) in intervention sites, CPAP in prior 5 years in 2708 (24.1%) in usual care sites and 600 (22.7%) in intervention sites, hypoglossal nerve stimulator in 147 (1.3%) in usual care sites and 38 (1.4%) in intervention sites, uvulopalatopharyngoplasty in 87 (0.8%) in usual care sites and 3 (0.1%) in intervention sites, oral mandibular device in 44 (0.4%) in usual care sites and 15 (0.6%) in intervention sites, and death within 7 days after discharge in 46 (0.4%) in usual care sites and 7 (0.3%) in intervention sites (eTable 1 in Supplement 2).

**Table 1.** Patient Characteristics

Characteristics	No. (%) of patients^a^	
	Usual care sites (n = 7454)	ASAP sites (n = 1747)
Age, y		
Mean (SD)	71.8 (10.8)	68.7 (11.1)
Median (range)	72 (22-107)	69 (27-100)
Sex		
Male	7114 (95.4)	1634 (93.5)
Female	340 (4.6)	113 (6.5)
Hispanic or Latino ethnicity	1012 (13.6)	92 (5.3)
Race		
Black	2243 (30.1)	744 (42.6)
White	4725 (63.4)	880 (50.4)
Other^b^	187 (2.5)	30 (1.7)
Unknown	299 (4.0)	93 (5.3)
Medical history		
TIA or stroke in prior year	1165 (15.6)	269 (15.4)
Hypertension	5996 (80.4)	1423 (81.4)
Hyperlipidemia	5206 (69.8)	1175 (67.3)
Diabetes	3232 (43.4)	745 (42.6)
Depression	2087 (28.0)	579 (33.1)
COPD	1480 (19.9)	354 (20.3)
Congestive heart failure	1198 (16.1)	335 (19.2)
Myocardial infarction	493 (6.6)	131 (7.5)
Peripheral vascular disease	1348 (18.1)	305 (17.5)
Atrial fibrillation	1041 (14.0)	214 (12.2)
Dementia	738 (9.9)	131 (7.5)
History of weakness	1379 (18.5)	307 (17.6)
Charlson Comorbidity Index, median (range)	1 (0-19)	1 (0-19)
Index event		
Stroke	5931 (79.6)	1429 (81.8)
TIA	1523 (20.9)	318 (18.2)
Full code status	6449 (86.5)	1606 (91.9)
Length of stay, median (range), d	3 (1-159)	3.6 (1-270)
Admitted to intensive care unit	644 (8.6)	179 (10.2)
NIHSS score, median (range)	2 (0-35)	2 (0-37)
90-d CAN mortality score, median (range)^c^	75 (0-99)	70 (0-99)
APACHE score, median (range)^c^	10 (0-50)	10 (0-42)

Abbreviations: APACHE, Acute Physiology and Chronic Health Evaluation; ASAP, Addressing Sleep Apnea Post-Stroke and Transient Ischemic Attack; CAN, Care Assessment Needs; COPD, chronic obstructive pulmonary disease; NIHSS, the National Institutes of Health Stroke Scale; TIA, transient ischemic attack.

^a^ Data are presented as number (percentage) of patients unless otherwise indicated.

^b^ Other races include American Indian and Alaska Native, Asian, and Native Hawaiian and Other Pacific Islander; race was self-identified in 99.7% of patients and was of unknown source in 10 patients.

^c^ APACHE is a measure of physiologic disease severity based on laboratory test data and vital signs.^51^ CAN score is a measure of mortality risk based on diagnoses and health care use.^46^

Patients at intervention and usual care sites were similar except for male sex (usual care: 7114 [95.4%]; intervention: 1634 [93.5%]; *P* = .02), depression (usual care: 2087 [28.0%]; intervention: 579 [33.1%]; *P* = .002), congestive heart failure (usual care: 1198 [16.1%]; intervention: 335 [19.2%]; *P* = .04), full code status (usual care: 6449 [86.5%]; intervention: 1606 [91.9%]; *P* = .02), 90-day CAN mortality score (usual care: median [IQR], 75 [55-90]; intervention: median [IQR], 70 [50-85]; *P* = .02), length of stay (usual care: median [IQR], 3.0 [1.9-5.7] days; intervention: median [IQR], 3.6 [2.0-6.9] days; *P* = .03), and age (usual care: mean [SD], 71.8 [10.8] years; intervention: 68.7 [11.1] years; *P* = .02) (Table 1). A history of weakness was more common during active implementation (baseline: 147 [15.4%]; active implementation: 138 [21.2%]; sustainability: 22 [15.2%]; *P* = .04) (eTable 2 in Supplement 2). Some differences in patient characteristics across intervention sites (eTable 3 in Supplement 2) and over time at usual care sites were observed (eTable 4 in Supplement 2).

### Primary Outcome: 30-Day OSA Diagnostic Testing Rate

**Figure 2.**
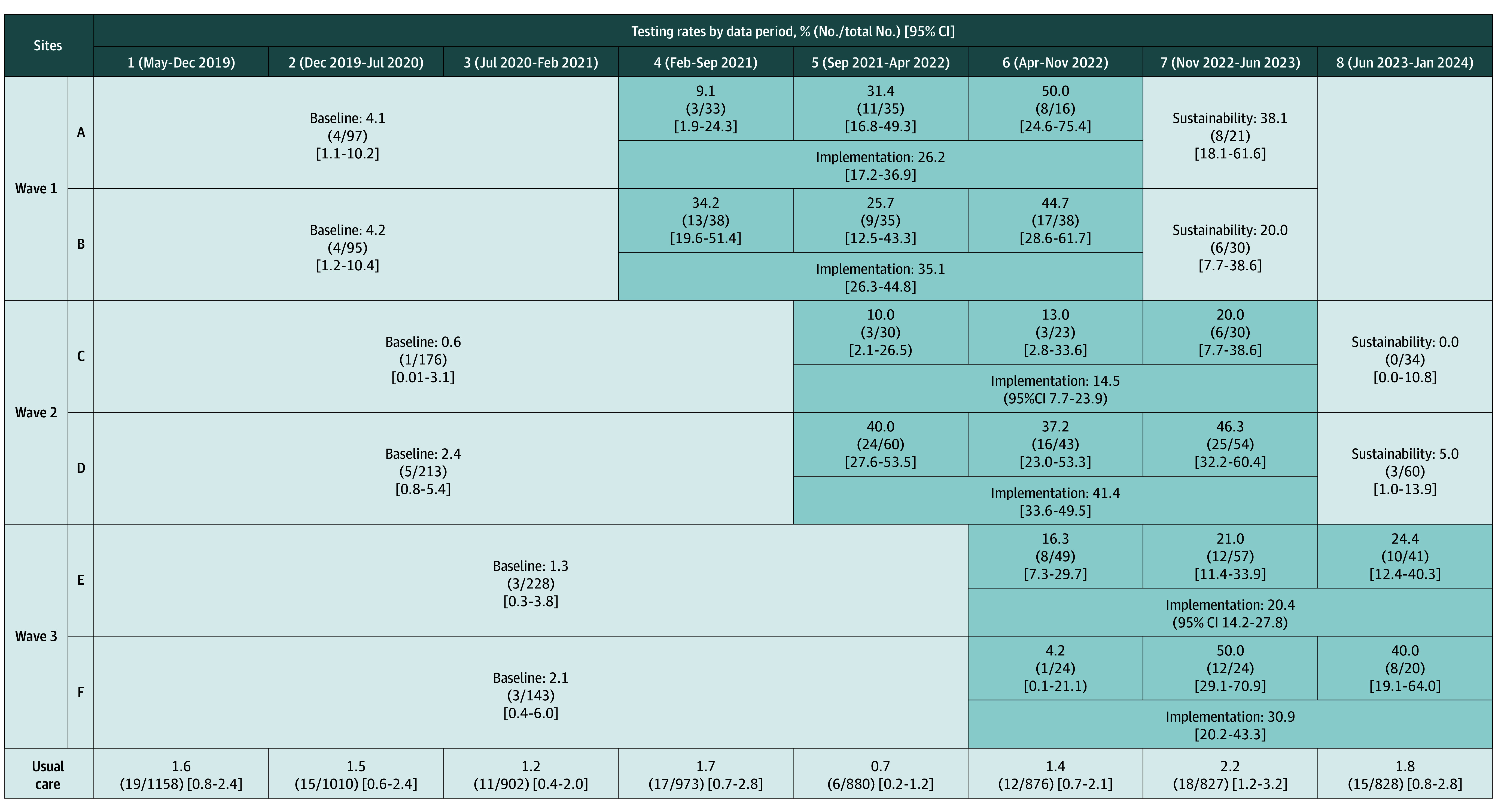
The 30-Day Sleep Apnea Diagnostic Testing Rates Across Study Phases Figure displays the sample size, duration (across 7-month data periods), and the 30-day sleep apnea diagnostic testing rate with 95% CIs for the 3 study phases—baseline, active implementation, and sustainability—for the intervention sites and for usual care sites across the study period. The primary analysis compared the 30-day sleep apnea diagnostic testing rate in the active implementation phase vs the baseline phase.

Thirty-day OSA diagnostic testing increased at intervention sites from 20 of 952 (2.1%; 95% CI, 1.3%-3.2%) during baseline to 189 of 650 (29.1%; 95% CI, 25.6%-32.6%) during implementation (Figure 2). Baseline OSA diagnostic testing rates varied across intervention sites from 1 of 176 (0.6%; 95% CI, 0.01%-3.1%) to 4 of 95 (4.2%; 95% CI, 1.2%-10.4%) and increased during active implementation from 12 of 83 (14.5%; 95% CI, 7.7%-23.9%) to 65 of 157 (41.4%; 95% CI, 33.6%-49.5%). The 30-day OSA diagnostic testing rate during sustainability was 17 of 145 (11.7%; 95% CI, 7.0%-18.1%). Thirty-day OSA diagnostic testing rates varied 6 of 880 (0.7%; 95% CI, 0.2%-1.2%) to 18 of 827 (2.2%; 95% CI, 1.2%-3.2%) at usual care sites across data periods.

Thirty-day OSA diagnostic testing rates increased during the 3 active implementation data periods; for example, for site A, the rate increased from 3 of 33 (9.1%; 95% CI, 1.9%-24.3%) to 11 of 35 (31.4%; 95% CI, 16.8%-49.3%) to 8 of 16 (50.0%; 95% CI, 24.6%-75.4%). Across intervention sites, the 30-day OSA diagnostic testing rate during the third active implementation data period was 74 of 199 (37.2%; 95% CI, 30.5%-44.2%). The unattended studies used by intervention sites were limited channel home sleep tests, usually NoxT3 (Nox Medical) or WatchPAT (Itamar Medical). Sleep studies at intervention sites identified sleep-disordered breathing in most patients (110 of 151 [72.8%) (eTable 5 in Supplement 2); 112 of 159 sleep studies (70.4%) were conducted during admission.

Primary and adjusted models revealed increased OSA diagnostic testing during active implementation vs baseline (unadjusted odds ratio [OR], 16.13; 95% CI, 8.25-31.53; adjusted OR [AOR], 16.90; 95% CI, 9.49-30.10) (Table 2). Increasing length of stay (AOR, 1.02; 95% CI, 1.01-1.03) and increasing stroke severity (NIHSS; AOR, 1.05; 95% CI, 1.02-1.08) were associated with higher 30-day OSA diagnostic testing. Although this study was not powered to evaluate changes in sustainability outcomes, 30-day OSA diagnostic testing was higher during sustainability vs baseline (OR, 3.36; 95% CI, 1.31-8.61; AOR, 3.58; 95% CI, 1.59-8.04).

**Table 2.** The 30-Day Sleep Apnea Diagnostic Testing Model Results

Variable	Primary model		Risk-adjusted model	
	OR (95% CI)	*P* value	AOR (95% CI)^a^	*P* value
Trial phase				
Active implementation vs baseline	16.13 (8.25-31.53)	<.001	16.90 (9.49-30.10)	<.001
Sustainability vs baseline	3.36 (1.31-8.61)	.02	3.58 (1.59-8.04)	.002
Data period				
2 vs 1	0.92 (0.50-1.69)	.78	0.90 (0.49-1.66)	.74
3 vs 1	0.82 (0.43-1.54)	.53	0.81 (0.43-1.52)	.51
4 vs 1	0.77 (0.43-1.36)	.36	0.76 (0.43-1.34)	.34
5 vs 1	0.74 (0.42-1.31)	.30	0.74 (0.42-1.30)	.30
6 vs 1	0.93 (0.53-1.61)	.78	0.93 (0.54-1.61)	.80
7 vs 1	1.66 (0.97-2.82)	.06	1.62 (0.95-2.75)	.08
8 vs 1	1.09 (0.60-1.98)	.77	1.07 (0.59-1.95)	.82
Intervention vs usual care sites	1.71 (0.85-3.44)	.13	1.59 (0.81-3.12)	.17
Covariates				
Length of stay, d	NA	NA	1.02 (1.01-1.03)	<.001
Neurologic symptom severity (NIHSS)	NA	NA	1.05 (1.02-1.08)	.001

Abbreviations: AOR, adjusted odds ratio; NA, not applicable; NIHSS, National Institutes of Health Stroke Scale; OR, odds ratio.

^a^ Multiple-imputation results.

### Secondary Outcomes

Thirty-day CPAP treatment rates were low across implementation sites (eTable 6 in Supplement 2) and increased from baseline (3 of /952 [0.3%]; 95% CI, 0.1-0.9) to active implementation (18 of 650 [2.8%]; 95% CI, 1.7-4.3) (Table 3). At usual care sites, 30-day CPAP treatment rates remained low across data periods, varying from 0.0% (0 of 876) to 0.4% (4 of 1158). The logistic model attained convergence for active implementation vs baseline (OR, 14.22; 95% CI, 2.40-84.40) (Table 3); however, the exact Wilcoxon signed-rank test comparing treatment between baseline and active implementation for intervention sites did not reach significance.

**Table 3.** The 30-Day Sleep Apnea Treatment Model (Primary Model) Results

Variable	OR (95% CI)	*P* value
Trial phase		
Implementation vs baseline	14.22 (2.40-84.40)	.009
Sustainability vs baseline	2.66 (0.13-56.21)	.48
Data period		
2 vs 1	0.40 (0.08-1.99)	.26
3 vs 1	0.64 (0.16-2.57)	.52
4 vs 1	0.39 (0.10-1.56)	.18
5 vs 1	0.40 (0.10-1.53)	.18
6 vs 1	0.23 (0.05-1.00)	.050
7 vs 1	0.60 (0.16-2.27)	.45
8 vs 1	0.27 (0.05-1.63)	.15
Intervention vs usual care sites	1.41 (0.31-6.33)	.64

Although results were in the expected direction, no statistically significant changes were observed in 90-day readmission or 90-day recurrent vascular event rates from baseline to active implementation (readmissions: OR, 0.92; 95% CI, 0.68-1.24; AOR, 0.89; 95% CI, 0.68-1.16; and recurrent vascular events: OR, 0.85; 95% CI, 0.56-1.29; AOR, 0.85; 95% CI, 0.60-1.22) (eTable 7 in Supplement 2).

### Exploratory Outcomes

At intervention sites, 90-day diagnostic testing increased from 36 of 952 (3.8%; 95% CI, 2.7%-5.2%) during baseline to 224 of 650 (34.5%; 95% CI, 30.8%-38.3%) during implementation. Similarly, 180-day diagnostic testing increased from 51 of 952 (5.4%; 95% CI, 4.0%-7.0%) during baseline to 250 of 650 (38.5%; 95% CI, 34.7%-42.3%) during implementation. Ninety-day treatment also increased from 7 of 952 (0.7%; 95% CI, 0.3%-1.5%) during baseline to 32 of 650 (4.9%; 95% CI, 3.4%-6.9%) during implementation. Similarly, 180-day treatment increased from 8 of 952 (0.8%; 95% CI, 0.4%-1.7%) during baseline to 36 of 650 (5.5%; 95% CI, 3.9%-7.6%) during implementation.

### Sensitivity Analyses

Findings similar to the main analyses emerged for 30-day OSA diagnostic testing rates (baseline vs active implementation) from sensitivity analyses adjusting for COVID-19 burden (AOR, 17.03; 95% CI, 8.66-33.49) (eTable 8 in Supplement 2) and for the Philips Respironics device recall (AOR, 16.38; 95% CI, 8.37-32.06) (eTable 9 in Supplement 2). In sensitivity analyses examining potential delays in establishing the intervention by removing the first active implementation data period, the active implementation effect was more robust than in the primary analysis (OR, 21.37; 95% CI, 10.34-44.19) (eTable 10 in Supplement 2).

## Discussion

In this cluster randomized trial of patients with acute cerebrovascular events, OSA diagnostic testing increased markedly at all intervention sites, supporting using QI to provide OSA testing for this population. Although many studies have investigated OSA management after cerebrovascular events, the ASAP trial contributes to the literature given its focus on evaluating a QI program implemented within the context of usual care. In other words, the ASAP trial was not conducted as a patient-level randomized clinical trial but rather as a site-level QI project.

Thirty-day diagnostic testing rate increases were achieved despite 3 major disrupting events: COVID-19 pandemic,^33^ Philips Respironics CPAP recall,^34^ and unprecedented fiscal strain at VA facilities.^52,53^ All 3 created substantial barriers to implementing QI activities focused on OSA management among patients with cerebrovascular disease (eg, competing demands on clinical staff, extraordinary staff shortages, and equipment scarcities).^54^ Despite these challenges, participating sites developed sleep medicine capacity to conduct sleep studies on inpatients.

As expected, adopting novel practices took time; at 4 of 6 sites, diagnostic testing rates in the first 7-month data period were lower than in later data periods. To increase diagnostic testing, sites had to implement processes to identify inpatients with stroke or TIA in near real time; create capacity to conduct inpatient sleep studies (typically unattended studies), and provide professional education to staff in sleep medicine and those caring for patients with cerebrovascular disease across wards and shifts. Establishing collaboration across traditionally siloed disciplines was challenging. Training neurology residents to order sleep studies required recurring educational efforts.

Thirty-day CPAP treatment rates at usual care sites and during baseline at intervention sites were low and increased only modestly during active implementation. Our primary outcome was the 30-day OSA diagnostic testing rate; therefore, QI activities focused on increasing sleep testing. A median length of stay of 3.5 days provided sufficient time for inpatient OSA diagnostic testing, but most patients were discharged shortly after the sleep study; therefore, treatment was primarily outpatient. Half of patients who received CPAP within 180 days after admission received it within 30 days, suggesting that losses to follow-up and delays in treatment were barriers to CPAP receipt. Only 21.8% of intervention site patients with a sleep study did not have sleep apnea, consistent with studies reporting that 70% of patients with stroke or TIA have OSA and highlighting the need to develop strategies to diagnose and treat OSA in this population.^1,2^ Facilities seeking to establish acute sleep medicine services should consider strategies to provide both OSA diagnosis and treatment during admission.

### Limitations

This study has key limitations that merit description. First, the ASAP trial was not powered to detect differences in secondary outcomes. Although we observed a statistically significant increase in 30-day CPAP treatment rates, treatment rates remained low. Hence, the ASAP trial could not examine associations between treatment and outcomes.^11^ Ongoing patient-level clinical trials (eg, Sleep SMART) may provide additional information about CPAP effects after stroke or TIA.^55^ Second, the ASAP trial was conducted within the VA, which has a culture of QI and a unified EHR system. OSA is highly prevalent among veterans; therefore, results may not generalize to other health systems. Third, we did not examine CPAP adherence. Fourth, in response to the COVID-19 pandemic and the Philips Respironics CPAP recall, sustainability was truncated, limiting analyses.

## Conclusions

In this cluster randomized trial study of patients hospitalized with acute ischemic stroke or TIA, QI activities increased 30-day OSA diagnostic testing. Future studies are needed to identify strategies to increase timely treatment among patients with acute cerebrovascular events who have OSA.

## Notes

Supplement 1.
Trial Protocol

Supplement 2. **eAppendix.** Imputation Details **eTable 1.** Exclusions from Cohort: Eligibility for Sleep Apnea Diagnostic Testing **eTable 2.** Intervention Site Patient Characteristics Over Study Phases **eTable 3.** Patient Characteristics Across Intervention Sites (N=6) **eTable 4.** Usual Care Site Patient Characteristics Over Study Data Periods **eTable 5.** Results of the Sleep Studies at Implementation Sites **eTable 6.** 30-Day Positive Airway Pressure (PAP) Treatment Rates at Intervention Sites **eTable 7.** Unadjusted and Adjusted Secondary Outcome Model Results by Study Phase **eTable 8.** Primary and Secondary Outcomes Adjusted for COVID-19 Burden **eTable 9.** Primary and Secondary Outcomes Adjusted for Restricted Access to Positive Airway Pressure Devices **eTable 10.** Primary and Secondary Outcome Model Results Excluding First Implementation Data Period
eReferences

Supplement 3.
Nonauthor Collaborators

Supplement 4.
Data Sharing Statement
