# Supplementary material for: Quality Improvement Intervention to Increase Sleep Apnea Diagnostic Testing After Stroke and Transient Ischemic Attack: A Cluster Randomized Trial
Source: JAMA Netw Open. 2025 Nov 14;8(11):e2543385. doi: 10.1001/jamanetworkopen.2025.43385 (PMC12619104; doi:10.1001/jamanetworkopen.2025.43385)
Supplement: Supplement 1. — Trial Protocol [file jamanetwopen-e2543385-s001.pdf]

Protocol Number: 19-12 / **1612509**

Title: Addressing Sleep Apnea Post-stroke (ASAP)

Funding Agency: Veterans Affairs HSR&D

Principal Investigators/Study Chairs: Jason J. Sico, MD, MHS & Dawn M. Bravata, MD

Date / Version: August 27, 2024; Version 17

ABSTRACT

BACKGROUND Approximately 11,000 Veterans present to a VAMC annually with an acute ischemic stroke or TIA.[1] The cornerstone of secondary stroke/TIA prevention includes delivering timely, guideline-concordant vascular risk factor management.[2] Over the past decade, obstructive sleep apnea (OSA) has been recognized as a potent, underdiagnosed, and inadequately treated cerebrovascular risk factor.[2,3] OSA is very common among patients with stroke/TIA with a prevalence of 70-80%.[4-16] Despite being highly prevalent, 70-80% of patients with OSA are neither diagnosed nor treated.[17] Untreated OSA has been associated with poor outcomes among patients with cerebrovascular disease including higher mortality and worse functional status.[11,18-21] The mainstay of OSA therapy is positive airway pressure (PAP).[22-24] PAP reduces recurrent vascular events, improves neurological symptoms and functional status among stroke/TIA patients with OSA.[2,15] The evidence favoring neurological recovery is strongest when interventions are applied early post-stroke/TIA. Guidelines recommend diagnosing and treating OSA for eligible stroke and TIA patients; however, within VHA, very few stroke or TIA patients receive OSA screening.[2,25] This guideline recommendation was informed in part by clinical trials utilizing an acute OSA assessment protocol developed and implemented by our group.[14,15]

OBJECTIVES: Utilizing a Hybrid Type I, randomized, stepped-wedge trial at 6 diverse VAMCs, to implement and evaluate the effectiveness and sustainability of implementation strategies utilized in an Addressing Sleep Apnea Post-stroke (ASAP) quality improvement program designed to improve facility-level diagnosis and management of OSA.

METHODOLOGY: We will initiate the ASAP program at 6 VAMCs that annually care for at least 50 Veterans admitted with a stroke but without a known history of OSA for a twenty-one-month intervention period followed by a sustainability period at 4 of the 6

participating sites. Effectiveness of the intervention will be measured across one primary effectiveness outcome (30-day facility-level diagnostic rate) and three secondary domains (30-day treatment rate, 90-day recurrent vascular event rate, and 90-day all-cause readmission rate). The ASAP quality improvement intervention will include a systems redesign virtual collaborative and data monitoring; implementation strategies will include external facilitation and audit and feedback. The Consolidated Framework for Implementation Research (CFIR) will be used to evaluate the implementation of the intervention and of the implementation strategies at several discrete time points during ASAP: (1) baseline; (2) active implementation, (7 Months, 14 Months, and 21 months, which marks the end of active implementation); and (3) at the end of the sustainability period. GO Scores and CFIR construct scoring will be completed for Wave 1 and Wave 2 sites across these time points. CFIR and GO Scores for Wave 3 sites will not include sustainability assessments; given COVID-related changes to the project timeline, Wave 3 sites do not have a sustainability period. The primary implementation outcome will be obtaining a GO score of 6 or more by the end of active implementation. The secondary implementation outcome will be a positive change in GO score of 4 or more by the end of active implementation. We will construct a business case analysis at the facility-level, including financial components related to the intervention. We believe that we can implement a sustainable ASAP program across diverse VAMCs which: (1) improves OSA diagnosis, treatment, and 90-day recurrent vascular event rate, and; (2) has a business case favoring intervention sites with the highest baseline recurrent vascular event rate.

CLINICAL RELATIONSHIPS: Interventions that improve diagnosis and management of OSA among Veterans with stroke/TIA are necessary to improve outcomes; implementation science strategies can assess the uptake and sustainability of this intervention. Because the intervention and implementation strategies have been designed using existing VHA infrastructure, if effective, the program would be ready for scaling system wide. Results from the business-case analysis for ASAP will be shared with VAMC providers and local leadership who may be interested in implementing this program at their facility. Additionally, an acute sleep service could be adapted to other patient populations (e.g., chronic obstructive pulmonary disease), both within and outside of VHA.

List of Abbreviations

ACS	Acute Coronary Syndrome
AHA/ASA	American Heart Association/American Stroke Association
ASAP	Addressing Sleep Apnea Post-stroke/TIA
BS	Bachelor of Science

CCSH	Certificate in Clinical Sleep Health
CDW	Corporate Data Warehouse
CHERP	Center for Health Equity Research and Promotion
CAN	Coincidence Analysis
CCMs	Configurational Comparative Methods
CHF	Congestive Heart Failure
CFIR	Consolidated Framework for Implementation Research (CFIR)
COVID	Corona virus disease
CPAP	Continuous Positive Air Pressure
DMAIC	Define, Measure, Analyze, Improve, and Control
DO	Doctor of Osteopathic Medicine
DSS	Decision Support System
CHAMP	CHAmpion Measure of Performance
FMEA	Failure Modes and Effects Analysis
FTE	Full Time Equivalent
GO	Group Organizational
HIPAA	Health Insurance Portability and Accountability Act
HST	Home Sleep Test
ICD	International Classification of Diseases
IOP	Intraoperative Platform
IRB	Institutional Review Board
LSI	Local Site Investigator
MBA	Master of Business Administration
MS	Master of Science
MSEd	Master of Science in Education

MD	Medical Degree
MIT	Medical Instrument Technician
MI	Myocardial Infarction
NP	Nurse Practitioner
OSA	Obstructive Sleep Apnea
ORCA	Organizational Readiness to Change Assessment
PAP	Positive Airway Pressure
PDSA	Plan, Do, Study, Act
PhD	Doctor of Philosophy
PRIS-M	Precision Monitoring to Transform Care
PRIUS	Prospectively-Reported Implementation Update and Score
PSAT	Program Sustainability Assessment Tool
PSG	Polysomnography
QUERI	Quality Evaluation Research Initiative
QI	Quality Improvement
R&D	Research and Development
REDCap	Research Electronic Data Capture
RPIW	Rapid Process Improvement Workshop
RPSGT	Registered Polysomnographic Technologist
RST	Respiratory Sleep Technologist
TIA	Transient Ischemic Attack
VA	Veterans Affairs
VAMC	Veterans Affairs Medical Center
VHA	Veterans Affairs Healthcare
VSSC	VHA Support Service Center

Contents

1. Introduction 6
2. Objectives 7
3. Study Procedures 9
   5.1 Study Design 9
   5.2 Recruitment Methods 13
   5.3 Informed Consent Procedures 15
   5.4 Inclusion/Exclusion Criteria 17
   5.5 Roles of the VA Staff and Veterans with Stroke/TIA and their Caregivers in ASAP 18
   5.5.c. Role of Veterans with Stroke/TIA and their Caregivers in ASAP 18
   5.6 Study Evaluations 18
   5.7 Data Analysis 23
      Control Sites 24
   5.7 Withdrawal of Subjects 34
4. Reporting 34
5. Privacy and Confidentiality 35
6. Communication Plan 37

1. Introduction

Approximately 11,000 Veterans present to a VAMC annually with an acute ischemic stroke or TIA.[14] The cornerstone of secondary stroke/TIA prevention includes delivering timely, guideline-concordant vascular risk factor management.[15] Over the past decade, OSA has been recognized as a potent, underdiagnosed, and inadequately treated cerebrovascular risk factor.[15, 16] OSA is very common among patients with stroke/TIA with a prevalence of 70-80%.[1, 2, 17-27] Despite being highly prevalent, 70-80% of patients with OSA are neither diagnosed nor treated.[28] History, physical examination, and screening instruments (e.g., Berlin Questionnaire) are inaccurate among patients with cerebrovascular disease.[41] Given that no combination of symptoms or physical examination findings has adequate sensitivity or specificity for the diagnosis of OSA post-stroke/TIA, only PSG or auto-PAP (used diagnostically) can identify disease status post-cerebrovascular event.[41] Recognizing these concerns, the AHA/ASA guidelines recommend that a sleep study (as opposed to screening questionnaires) should be used to identify the presence or absence of OSA among eligible patients with acute ischemic stroke or TIA.[15]

The American Academy of Sleep Medicine (AASM) recommends PSG either conducted in a sleep laboratory (the gold standard) or unattended PSG conducted in patients' homes for the detection of OSA.[42] Unfortunately, there are several barriers to the use of PSG. Within the VHA there are long wait times for scheduling PSG (within or outside of the VHA). Veterans in rural areas often must travel great distances to the nearest sleep laboratory. An alternative to PSG in a sleep laboratory is unattended PSG.[43] Studies have reported good agreement between unattended PSG and formal PSG (sensitivities: 75-100%; specificities: 87-100%).[43] The VHA Sleep-Related Breathing Disorders Source Book states that alternatives to full PSG may be acceptable when patients cannot be assessed with PSG in a sleep laboratory.[34] Other alternative approaches include the use of auto-PAP. Several studies have evaluated the use of auto-PAP devices used diagnostically, finding them to have acceptable validity among stroke/TIA patients.[2, 44]

Untreated OSA has been associated with poor outcomes among patients with cerebrovascular disease including higher mortality and worse functional status.[24, 29-32] The mainstay of OSA therapy is PAP.[33-35] The literature demonstrating the benefits of PAP among the general population with OSA is extensive. PAP therapy dramatically reduces or eliminates sleep-related breathing perturbations and improves a variety of outcomes including daytime sleepiness, cognition, quality of life and hypertension control.[45] Beyond these general benefits of PAP therapy for OSA, randomized clinical trials and observational cohort studies have demonstrated the effectiveness of PAP in improving neurological and functional outcomes post-stroke/TIA. Several randomized trials evaluated the use of early PAP in the acute stroke period. These acute stroke

studies have demonstrated improvements in neurologic symptoms with PAP therapy that are equivalent to those observed with tPA therapy.[2, 23, 46-48] One randomized trial evaluated the use of early PAP among acute TIA patients found that the vascular event rate decreased as PAP use increased: 8% for no PAP use; 6% for some PAP use; and 0% for good PAP use.[1] Four randomized controlled trials evaluated PAP in subacute stroke patients.[49-51] The largest of the cohort studies (N=189) reported that patients ≥2 months post-stroke with OSA who did not use PAP had much higher recurrent stroke rates than patients who used PAP (32% vs 14%, p=0.02) and higher adjusted incidence of nonfatal vascular events (HR 2.87 [1.11- 7.71]).[47] The number-needed-to-treat to prevent one new vascular event was 4.9 patients. A recent meta-analysis of ten randomized controlled trials among patients with ischemic stroke identified a relationship between neurological symptom improvement and PAP adherence.[12] A study of patients ≥6 weeks after an index stroke found that PAP users had a recurrent vascular event rate of 3% compared with 15% in PAP non-users.[13] These data demonstrate that recurrent event reduction occurs when treatment is provided early and when patients adhere to PAP.

In short, PAP reduces recurrent vascular events, improves neurological symptoms and functional status among stroke/TIA patients with OSA.[1, 15] The evidence favoring neurological recovery is strongest when interventions are applied early post-stroke/TIA. Guidelines recommend diagnosing and treating OSA for eligible stroke and TIA patients; however, within VHA, very few stroke or TIA patients receive OSA screening.[3, 15] This guideline recommendation was informed in part by clinical trials utilizing an acute OSA assessment protocol developed and implemented by our group.[1, 2] To address the observed gap in care, we propose a Hybrid Type I, randomized, stepped-wedge trial to increase the rate of timely, guideline-concordant diagnosis and treatment of OSA among Veterans with ischemic stroke/TIA and thereby reduce recurrent vascular events.

2. Objectives

Utilizing a Hybrid Type I, randomized, stepped-wedge trial at 6 diverse VAMCs as intervention sites and 30 usual care control sites, to implement and evaluate the effectiveness and sustainability of implementation strategies utilized in an Addressing Sleep Apnea Post-stroke (ASAP) program designed to improve facility-level diagnosis and management of OSA. These objectives will be accomplished through the following specific aims:

Specific Aim 1: Evaluate the effectiveness of an intervention for the early diagnosis and treatment of OSA among Veterans with ischemic stroke and TIA. The intervention program will include a systems redesign Virtual Collaborative and data monitoring. ASAP includes external facilitation and audit and feedback as implementation strategies, which will be assessed during the intervention period.[36] Sites will locally-tailor and implement an Addressing Sleep Apnea Post-stroke (ASAP) quality improvement program. The sites will choose a diagnostic strategy (i.e., unattended polysomnography [PSG]/home sleep test [HST], in-laboratory PSG, direct to auto-titrating [auto]-PAP) and a therapeutic strategy (e.g., in-laboratory PAP titration, auto-PAP). The primary effectiveness outcome, evaluated at the facility-level, will be the 30-day sleep apnea diagnostic rate. The three secondary effectiveness outcomes will be 1. the 30-day

treatment rate, 2. the 90-day recurrent vascular event rate and 3. the 90-day all-cause readmission rate. We hypothesize that the: mean facility diagnostic rate (PSG within 30 days of presentation) will be 70% for eligible patients during intervention periods vs 7% for controls; the mean facility treatment rate (PAP initiation within 30 days of presentation) will be 50% for intervention vs 2% for controls; mean facility 90-day recurrent vascular event rate (stroke, MI/acute coronary syndrome [ACS], congestive heart failure [CHF] and all-cause mortality) will be 3.5% for intervention vs 10% for controls; and; mean facility 90-day all-cause readmission rate will be 20% for intervention vs 33% for controls. Chart review and administrative data will be used to ascertain outcomes.

Specific Aim 2: Evaluate the implementation of the intervention program. This implementation evaluation will be grounded in the Consolidated Framework for Implementation Research (CFIR)[37-39] and examine how implementation strategies and features of local context combine and interact to influence the implementation of ASAP across six diverse VAMCs. We hypothesize that this project will contribute generalizable knowledge to implementation science through detailed specification and evaluation of each implementation strategy, in-depth characterization of local context at the six facilities, and rigorous qualitative and mixed-methods analyses to examine how implementation strategies and features of local context influence outcomes. Furthermore, we hypothesize that contextual factors will be associated with our implementation outcomes, both primary (i.e., obtaining a GO score of 6 or more by the end of active implementation) and secondary (i.e., a positive change in GO score of 4 or more by the end of active implementation).The analyses leading to these findings will focus both within and across facilities, with special attention to facility- and patient-level factors contributing to implementation success. CFIR constructs will be applied to de-identified interview data.

Specific Aim 3: Evaluate the sustainability of the intervention. Sustainability will be evaluated immediately following the 21-month active implementation period and will be of variable duration based on WAVE randomization (WAVE 1 and 2 sites will have each have a 7-month sustainability period whereas WAVE 3 will not have a sustainability period). The primary sustainability outcome at WAVE 1 and 2 sites will include the number of patients who receive diagnosis within 30 days of presentation. We hypothesize that sustainability will be greatest at sites that use HST as their primary diagnostic approach and have champions who continue to engage with systems redesign.

Specific Aim 4: Perform a business-case analysis. We will construct a business-case analysis at the facility-level and will include financial components related to the intervention. We hypothesize that the business-case analysis will favor the intervention at sites with the highest baseline recurrent vascular event rate.

The significance of the proposed ASAP project to the VHA is grounded in both the high prevalence of OSA among Veterans and the low baseline rates of guideline concordant OSA screening post-stroke/TIA. This proposal addresses two major barriers in the implementation of the AHA/ASA OSA screening guideline: lack of a study powered to detect differences in recurrent vascular events and lack of a business-case analysis to support adoption of the AHA/ASA recommendation. If the project improves screening

and treatment of OSA, then it is likely that the Veteran population will realize significant improvements in clinical outcomes. The planned implementation evaluation and sustainability assessment will provide insights that are likely to be relevant to other quality improvement (QI) activities. The ASAP proposal advances the priorities of our partners within VA Patient Care Services (Drs. Sharyl Martini, Glenn Graham, and Claibe Yarbrough). Moreover, the evaluation of an "acute sleep service" is an innovative approach that is generalizable both within and outside VHA. The intervention and implementation strategies have been designed using existing VHA infrastructure, so if the intervention is effective, then the program will be ready for scaling system wide.

3. Study Procedures

5.1 Study Design

5.1.a. ASAP Overview The ASAP project will be conducted as a Hybrid Type I, randomized, stepped-wedge trial at 6 intervention sites and 30 control sites over a 3.5-year period (Figure 1). The 6 intervention sites will begin the 21-month active implementation period; (orange shading for WAVE 1, green shading for WAVE 2 and yellow shading for WAVE 3) in 3 staggered "steps" with 2 sites per step. Immediately following the active implementation phase for WAVES 1 and 2 will be a sustainability phase up to 7 months in duration (purple shading). WAVE 3 will not have a sustainability phase. This design allows for 3 active implementation data periods of 7-months duration for each WAVE. As seen in Figure 1, there are a variable number of 7-month data periods in the baseline and the sustainability periods by wave. The ASAP quality improvement intervention includes a systems redesign Virtual Collaborative and data monitoring. As part of the quality improvement intervention, sites will locally tailor and implement an ASAP approach, in which each site chooses a diagnostic strategy from a menu that includes unattended PSG, in-laboratory PSG, or direct to auto-PAP approach. Similarly, sites may choose any treatment strategy.

Figure 1. ASAP Project Active Implementation and Sustainability Timeline

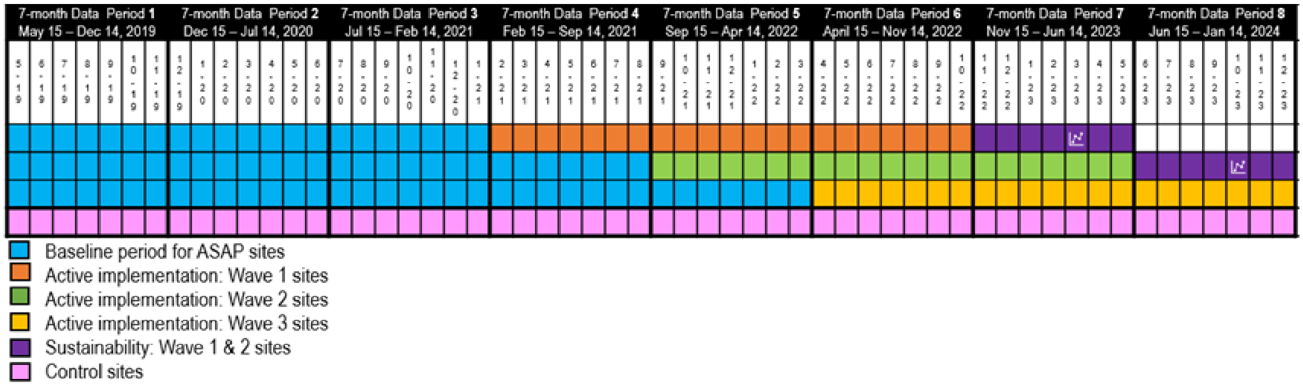

5.1.b. Participating Sites To be eligible for inclusion in ASAP a site had to have >50 stroke/TIA patients per year and have at least 1.0 FTE staff dedicated to systems redesign. The sites were chosen because they are diverse in terms of geography and sleep infrastructure. Data regarding the capacity to perform acute OSA assessment at each site is included in site investigators' letters of support. Furthermore, the sleep clinical champions vary across the sites and include clinicians trained in pulmonary, neurology, and psychiatry; some have on-site sleep laboratories whereas others use unattended PSG or fee-basis for their PSGs.

Table 2 data provide the estimates that were used in the revised power calculations: 111 patients per year will be eligible.

Table 2. Baseline Characteristics of ASAP Intervention Sites

VAMC (Site)	Annual Patient Volume (n) [a]	History of OSA (n)	PSG in prior 5-years (n)	Potentially Eligible (n) [b]
A	212	30	18	172
B	150	26	17	111
C	169	52	20	107
D	157	30	23	116
E	141	37	37	92
F	172	39	32	117
National average	158.2	37.3	25.4	107.5
**ASAP sites**	**157.3**	**35.2**	**20.3**	**119.2**

[a]Annual stroke/TIA volume based on FY2017 data and excludes patients with a history of OSA. [b]Potentially eligible patients exclude those with: history of OSA, PSG in prior 5 years, death within 7-days of discharge, and hospice referral.

5.1.c. Intervention Description The quality improvement intervention will include: a systems redesign Virtual Collaborative and data monitoring.

Systems Redesign Virtual Collaborative A Virtual Collaborative will be convened among the investigators and the clinical team members from the active implementation sites. The site clinical teams will include members from the stroke team, sleep medicine, and systems redesign. The Virtual Collaborative will be used to develop system redesign approaches to overcome barriers to

implementation, share lessons and resources across sites, and foster a sense of community around stroke and sleep care. The Virtual Collaborative will be conducted using a virtual kick-off event, followed by monthly video-teleconferencing. As part of the kick-off event, Dr. Sico, a Lean Six Sigma-trained Black Belt, will lead a mini-rapid process improvement workshop (RPIW). The site teams will: (1) map the local current value stream; (2) facilitate team problem solving; (3) design an achievable future state process map (including the specific approach to diagnosis and treatment that will be used at the site), and; (4) begin early discussions regarding sustainability of the intervention. Define, Measure, Analyze, Improve, and Control (DMAIC) is a prescribed improvement process for Systems Redesign/Lean Six Sigma methodology which focuses on identifying and defining root causes of a problem prior to applying solutions, implementing solutions linked to an underlying cause, and then establishing best practices to assure that an intervention is effective and sustainable. DMAIC utilizes such techniques as process mapping, PDSA matrices, and Failure Modes and Effects Analysis (FMEA). Together, these approaches help staff systematically identify and remove non-value-added work to the process and develop breakthrough projects which remove defects in the process.[59, 60]

ASAP will leverage an existing national platform called the Integrated Operations Platform (IOP), which includes several components that can be used for service planning; it is a web-based tool that is openly accessible (no passwords required). The IOP has successfully fostered collaborations across VAMCs. It has provided a platform for VA leadership to identify best practices and “push” strategies. The IOP hub provides a dynamic learning environment that supports the theme of promoting a learning healthcare system. The IOP hub will serve the ASAP project as a connectivity hub for investigators, site PIs, systems redesign personnel, and VHA operational partners. Resources including staff education materials, articles, protocols, and project charters will be shared in the library.

We will utilize VA Research Electronic Data Capture (REDCap) database monthly reflection survey, the Prospectively-Reported Implementation Update and Score (PRIUS), data collected from monthly ASAP Collaborative Calls (e.g., triumphs, barriers, and goals reported by sites, field memos), to obtain monthly updates on site specific processes and procedures, which will then be used by the ASAP Implementation Team to inform External Facilitation with the sites while promoting the development of a broader ASAP Community of Practice across sites. Active intervention site team members will participate in monthly collaborative virtual conference calls. During these calls, sites will share lessons learned as they implement the action plans that were developed during the kick-off, brainstorm together about overcoming barriers to ASAP implementation, review interim data, and support likeminded VHA staff/providers who are all working to improve care and outcomes for Veterans with stroke/TIA. Study staff will take notes during the call. Furthermore, sites will submit slides regarding what has worked well during the last month (i.e., facilitators), areas of improvement (i.e., barriers), and goals for the upcoming month. Information collected during the calls will be used by the Implementation Team to inform

future External Facilitation calls and discussions related to CFIR constructs and GO score scoring. Text from the PRIUS calls, ASAP collaborative calls, External Facilitation call interactions, will be uploaded to NVivo for analysis by the team.

Data Monitoring Chart review will be conducted centrally (Indianapolis VAMC/West Haven VAMC) and at the local sites. Chart review will be conducted on all eligible ischemic stroke and TIA patients who are admitted at the participating sites. Patients with ischemic stroke/TIA will be identified using ICD-10 codes in the Corporate Data Warehouse (CDW). The hub will include facility-level data for: OSA diagnosis and ASAP primary and secondary effectiveness outcomes. The hub allows sites to compare their data to other sites and to examine trends over time. Site staff/providers will use these data to iteratively improve ASAP implementation. For example, if sites learn that OSA testing rates are lower after discharge then during the hospitalization period, processes focusing on hospitalized (rather than discharged) patients would be appropriate. To compliment data seen on the Hub, the results of the chart review will be provided to implementation sites and will be discussed at the Virtual Collaborative monthly conferences. Furthermore, recognizing that each patient is an opportunity to improve local ASAP processes and procedures, during external facilitation sessions, patient-level data will be reviewed with the sites.

5.1.d. Implementation Strategy This project draws explicitly upon the implementation strategies of external facilitation and data and feedback, which will be assessed on an ongoing basis over the intervention period.[36, 61-63]

External Facilitation External facilitation changes over time based on the needs of the team.[65] However, the core elements of relationship-building, methods training, communication, facilitating team-based problem solving, and monitoring performance over time will be preserved through external facilitation from a Lean Six Sigma Black Belt experienced in stroke QI work and ASAP investigators experienced in implementation of post-stroke/TIA OSA programs.[85]

Audit and Feedback[63] As described above, active implementation sites will receive monthly feedback on quality indicators for ASAP-related process and outcome data. To support the sites' use of data, the local teams will receive explicit training in team-based reflecting & evaluating, goalsetting, and planning. Meeting as a group, local teams will be encouraged to formally ask and answer questions like: "How are we doing?" "Are we where we want to be?" "What performance goals do we want to set as targets?" "What do we need to do to achieve our goals?" "How will we know how far or how close we are to hitting our targets?" The audit and feedback implementation strategy is a key element of the ASAP approach to sustainability, as the activities of reflecting & evaluating, goal setting and planning allow local teams to engage in continuous improvement, stepping back as a group on a periodic basis to review data related to the trajectory of their progress over time, make adjustments as needed to address any dips in performance, and consider how to sustain improvement. Hub usage data will also be collected and summed by site in order to determine how often each month the hub is accessed by each site to audit their own performance.

5.1.e. Site Randomization As described above, the ASAP study will be conducted as a stepped-wedge trial with six sites randomly allocated to receive the intervention in three steps, two facilities at a time. At the start of the study, the order in which sites will receive the ASAP program will be derived from a computer-generated randomization list created by ASAP senior statistician (Dr. Joanne Daggy).

5.2 Recruitment Methods

5.2.a General Recruitment Overview As the quality improvement intervention is applied at the facility-level, rather than at the patient-level, the primary unit of analysis for ASAP is at the facility-level. Therefore, given that all six sites have already agreed to participate, recruitment has been completed. The primary effectiveness outcome, evaluated at the facility-level, will be the proportion of patients at each site which undergo and complete diagnostic testing (30-day sleep apnea diagnostic rate) For this rate, the denominator includes all eligible patients at the participating facilities. Because the ASAP program focuses on providers and systems of care, patients will not be consented. Each local site R&D board will be requested to provide approval for research to take place at that location.

ASAP sites will change the standard practice for all eligible ischemic stroke and TIA patients such that the routine clinical experience will be to receive OSA diagnosis and treatment. In the same way that all ischemic stroke/TIA patients receive other guideline-recommended care, patients at ASAP sites will also routinely receive OSA diagnosis and treatment. Certainly, our goal is to encourage all eligible patients to accept the recommendation to receive OSA diagnosis and treatment and the degree of patient acceptance will be a key component of the implementation evaluation, however the concept of recruitment as would be applied in a patient-level clinical trial is not applicable to this facility-level stepped-wedge trial. Similarly, although a patient-level trial would include a detailed description related to participant selection, for this facility-level analysis, patient selection is considered in terms of the patients who will be included in the denominator for each of the effectiveness outcomes as described above. However, the treating provider will determine if any given patient has a contraindication to PAP.

For Health Care Providers, we will request a waiver of documented consent (for interviews). We will record a verbal acknowledgement that the interviews are being recorded. No Health Insurance Portability and Accountability Act (HIPAA) information is being requested or accessed.

For Veteran patient chart reviews, we will request a waiver of consent and a waiver of HIPAA authorization. This study implements standard of care treatment for all patients. We will request a waiver of documented consent for Veteran interviews.

5.2.b. Procedures and Recruitment related to Specific Aim 2 (Implementation Assessment)

VA Employees Dr. Sico and one other member of the research team will conduct, if permissible, on-site, in-person baseline site visits at each facility (green shading, Figure 1) In preparation for challenges posed by COVID 19, the team may forgo in-person baseline visits and may perform the baseline visit, virtually (using video and teleconferencing), if necessary. Individual semi-structured interviews will be conducted with champions and staff/providers who are involved in stroke/TIA patient care, likely including: members of the stroke team (usually neurology and nursing), sleep medicine, the hospitalist service, primary care, and systems redesign. A paper version of the interview will be used to accommodate employees who, because of language limitations, prefer to respond to a written document. We will contact local Service Chiefs and union leadership for permission to interview employees and assist in identifying key informants. We will contact the Medical Center Director prior to site kick-offs. We will ask permission of Service Chiefs to have their staff/providers excused from clinical/administrative responsibilities, especially for the purposes of the kick-off, be it in-person or virtually, interviews and kick-off using methods successfully implemented for similar projects.

Veteran Recipients of local ASAP/acute OSA testing and treatment quality improvement initiatives Local QI initiatives are being designed and implemented with the intention to improve care quality and delivery for Veterans who have survived an ischemic stroke or TIA. We seek to conduct interviews to gain the Veteran and caregiver perspective on their experience with being the recipient of a local ASAP QI initiative, as well as identifying potential areas of improvement.

As such, we will be sending letters via USPS and MyHealtheVet to Veterans who have been participating in local ASAP initiatives to invite them to participate in an interview. The letter will ask the Veteran to call the ASAP research staff if they do not wish to participate or if they have any questions about the project. The letter will state that the ASAP staff will begin calling to schedule interviews 2 weeks after the date on the letter. We will make no more than 3 attempts by telephone to contact the Veteran to schedule the interview. A project information sheet will be included in the letter, so the Veteran understands the risks and benefits of participating. Recorded verbal consent will be obtained from each person (provider, employee, or Veteran) being interviewed. Interviews will take less than one hour.

5.2.c. Procedures and Recruitment related to Specific Aim 3 (Sustainability Assessment) Similar to our approach in the Specific Aim 2 Implementation Analysis, we will ask permission of Service Chiefs and union leadership to request employees to complete surveys and participate in interviews. Among WAVE 1 and 2 sites, we will ask Service Chiefs to have their staff/providers excused from clinical/administrative responsibilities to participate in the sustainability assessment occurring near the end of a site's sustainability period. Sustainability will be assessed via surveys and interviews. Immediately prior to the start of the sustainability period (Figure 1, purple shaded area), ASAP end users (including the site ASAP champion and members of the team locally implementing the ASAP protocol) and other personnel included on the team

project charter will be asked to participate. Consent will be obtained from each subject. Interviews will take less than one hour. We have successful experience as VA investigators with one-year follow-up interviews on other VA projects. We have used a trio of engagement strategies that have proven effective. At both the baseline and one-year intervals, we use a semi-structured guide that assists interviewers in building rapport with participating VA staff/providers while asking questions on a standardized set of topics. This provides opportunities for participating staff/providers to “feel heard” when sharing their perspectives. Furthermore, we always write individualized and tailored thank-you messages to every participant within a week of the interview. Finally, we are extremely flexible in terms of scheduling interviews. Of note, while WAVE 3 sites do not have a formal sustainability period, we will include as part of their active implementation interview (conducted at the end of active implementation) questions related to sustainability of ASAP.

5.3 Informed Consent Procedures

Overview of Informed Consent Procedures Staff/providers involved in the care of acute stroke and TIA patients as well as staff/providers from the sleep medicine and respiratory therapy services will be approached for participation in the program and for participation in the interviews. Each local site R&D board will be requested to provide approval for research to take place at that location.

Interviews with VHA Employees Chief of staff, appropriate service chiefs (e.g., Sleep Medicine, Neurology), and union leadership will be contacted for approval to interview staff/providers at their facilities. VHA staff/providers members who participate in the interviews will be provided with an information sheet regarding the study. Verbal consent will be obtained from staff/providers for recorded interviews and standardized assessments. Consented staff/providers will be given ample opportunity to inquire about details of the study prior to agreeing to participate in any of the interviews or assessments. No individually identifying information will be collected via the interviews and will only be labelled with a code number that is not derived from any identifying information. It is noted, however, that if identifying information is stated in the interview, the final transcript will be de-identified.

After giving verbal consent, we will discuss implementation of the ASAP protocol, their perception of barriers and facilitators to implementing the intervention, and examine their opinions related to the implementation strategies associated with the ASAP protocol. As described above, no individually identifying information will be collected via the interviews and interview tapes and transcripts will only be labelled with a code number that is not derived from any identifying information. Any potentially identifying information stated in the interview will be de-identified from final transcript which will serve as the data source for the qualitative analyses. Furthermore, to mitigate concerns regarding divulging potentially negative information regarding their individual site(s) or management, we will review the process by which the ASAP team will maintain their confidentiality, assuring all subjects that their confidentiality will be maintained throughout the study and that specific data that an individual subject shares with ASAP

personnel will not be shared with any other facility personnel. No HIPAA protected information will be requested of the VA staff/providers and administrators.

Surveys Completed by VHA Employees The Local Site Investigator (LSI) and ASAP Field Staff will be sent a REDCap survey near the end of each calendar month to reflect on their team's experience with ASAP in that month. This includes barriers to implementing ASAP, how they have overcome these barriers, and diagnostic and treatment modalities available in their VAMC. The surveys all say, "Voluntary, confidential and anonymous" but we aren't necessarily getting consent.

Intervention participation Chief of staff appropriate service chiefs (e.g., Sleep Medicine, Neurology), and union leadership will be contacted for approval for providers to participate in the ASAP proposal. An information sheet will be provided to VHA employees prior to their enrollment in the intervention.

Interviews with Veterans Veterans admitted with an ischemic stroke or TIA will be contacted by USPS or MyHealtheVet to invite them to participate in an interview. Veterans who participate in the interviews will be provided with an information sheet regarding the study. Verbal consent will be obtained and digitally recorded from the Veteran for a recorded interview. Veterans agreeing to be approached will be given ample opportunity to inquire about details of the study prior to agreeing to participate in the interview. No individually identifying information will be collected via the interviews and will only be labelled with a code number that is not derived from any identifying information. It is noted, however, that if identifying information is stated in the interview, the final transcript will be de-identified.

After giving verbal consent, we will discuss the Veteran's experience with the ASAP protocol, their perception of barriers and facilitators to participating in the intervention, and examine their opinions related to the experiences associated with the ASAP protocol. As described above, no individually identifying information will be collected via the interviews and interview recordings and transcripts will only be labelled with a code number that is not derived from any identifying information. Any potentially identifying information stated in the interview will be de-identified from final transcript which will serve as the data source for the qualitative analyses. Furthermore, to mitigate concerns regarding divulging potentially negative information regarding their experiences, we will review the process by which the ASAP team will maintain their confidentiality, assuring all subjects that their confidentiality will be maintained throughout the study and that specific data that an individual subject shares with ASAP personnel will not be shared with any other facility personnel. No HIPAA protected information will be requested of the Veterans.

Chart Review For Veteran patients, we will request a waiver of HIPAA authorization and informed consent, such that chart review can be performed to examine demographics, past medical history, and whether the Veteran received guideline concordant neurological care. Chart review will be conducted on all eligible patients admitted to a facility with the diagnosis of ischemic stroke and

TIA (please refer to inclusion/exclusion criteria as noted in section IA), regardless of whether they receive PAP therapy. Chart review data will be merged with electronic health record data (e.g., from the CDW). The CDW algorithms will be iteratively improved based on chart review findings. Phone numbers and addresses of Veterans will be abstracted from chart review. See HIPAA Form 103 for additional details.

5.4 Inclusion/Exclusion Criteria

As noted in Section 5.2.a, General Recruitment Overview, recruitment is at the facility-level and includes VHA employees, rather than patients.

To have been an eligible facility for ASAP, the VAMC would have had to have >50 stroke/TIA patients per year and have at least 1.0 FTE staff dedicated to systems redesign.

Within these VAMCs, VHA employee subjects will be the primary targets of this research project. Inclusion criteria include employee subjects who either have direct involvement in the management of Veterans admitted to a VA Medical Center (VAMC) with a cerebrovascular event, either stroke or TIA, (e.g., neurologists, nurses) or in the evaluation and management of OSA (e.g., sleep medicine physicians, respiratory and sleep technologists) or in process improvement work (e.g., systems redesign personnel, administrators). Exclusion criteria include employee subjects who are neither caring for Veterans admitted for a cerebrovascular event nor involved in the evaluation and management of OSA nor are involved in process improvement work. Our target recruitment for VA employee subjects is up to 30 subjects per ASAP site.

Veterans will be included in the research in terms of chart review and interviews but will not be a direct target of the intervention. Veterans included in the research will be those admitted to an ASAP VAMC with the diagnosis of ischemic stroke or TIA who do not have an existing diagnosis of sleep apnea. Chart review will be conducted on all patients meeting this inclusion criteria. Veterans who have been the recipients of local ASAP protocols will be invited for interviews. We will interview 5 Veterans per site and no more than 30 Veterans in total, at which time we expect to reach thematic saturation.

In considering the types of Veterans who would be eligible for sleep apnea diagnostic testing or treatment, we should first note that at all times treating providers (the target of the research) will hold patient safety in the highest regard. Thus, by keeping contraindications to PAP therapy in mind, providers may decide to exclude patients from PAP therapy. This would be the case for Veterans who meet inclusion criteria for the study (i.e., being admitted to an ASAP VAMC with the diagnosis of ischemic stroke or TIA who do not have an existing diagnosis of sleep apnea) but their healthcare provider, in the course of delivering routine clinical care, makes the decision that a given Veteran has a contraindication to PAP therapy. Inclusion criteria would include patients admitted to an ASAP VAMC with the diagnosis of an ischemic stroke/TIA without a prior diagnosis of OSA. Exclusion criteria for Veterans will include being admitted to an ASAP VAMC with a diagnosis other than ischemic stroke or TIA (e.g., syncope, seizure), Veterans not admitted to a VAMC for their acute cerebrovascular care

(e.g., presented to the Emergency Department and transferred to a non-VAMC), or a known history of sleep apnea.[26]

5.5 Roles of the VA Staff and Veterans with Stroke/TIA and their Caregivers in ASAP

5.5.a. Roles of the LSI and Field Staff Each LSI will serve three roles in the ASAP study: (1) subject; (2) clinical provider conducting local QI projects to improve diagnosis and treatment of OSA among stroke/TIA patients, and; (3) member of the ASAP research team.

Subject Each LSI/Field Staff will participate in both interviews and completing surveys.

Conducting QI Each LSI/Field Staff will serve as a local champion and conduct QI projects designed to improve diagnosis and treatment of OSA among Veterans with stroke/TIA. This will include participating in the site kick-off, using the IOP, and engaging with the ASAP research team

Member of the ASAP research team: Each LSI/Field Staff will also serve to recruit other local personnel to participate in interviews and surveys.

5.5.b. Role of VA Staff who are neither an LSI nor Field Staff

Subject: VA staff/providers who are actively participating in conducting QI initiatives related to ASAP or engaged partners/key stakeholders identified by the LSI/Field Staff will participate in interviews. This group will not participate in surveys.

5.5.c. Role of Veterans with Stroke/TIA and their Caregivers in ASAP

Subject: Veterans admitted to a VAMC during active implementation with an ischemic stroke or TIA and their caregiver(s) will participate in interviews. This group will be identified by the LSI/Field Staff. This group will not participate in surveys.

5.6 Study Evaluations

The data sources for ASAP include:

(1) Interviews with:

a. Staff conducting QI initiatives for ASAP
b. Veterans/caregivers admitted to a VAMC during ASAP active implementation

(2) Surveys and standardized assessments completed by staff:

a. Organizational Readiness to Change Assessment (ORCA)
b. Program Sustainability Assessment Tool (PSAT)
c. Monthly REDCap assessment completed by LSI/ASAP Field Staff

(3) Assessments completed by ASAP research team:

   a. Champion Measure of Performance (CHAMP) measure
   b. Prospectively-Reported Implementation Update and Score (PRIUS)
   c. Group Organizational (GO) Score
   d. Consolidated Framework for Implementation Research (CFIR) coding

(4) Notes and debriefs from interactions the ASAP research team has with champions and others involved in local implementation of QI ASAP initiatives, including those collected from external facilitation contacts and monthly Collaborative Calls

(5) Chart review from ASAP patients and patients at control sites

(6) Administrative data (i.e., Corporate Data Warehouse [CDW] data, VA vital status files, VHA Support Service Center [VSSC], Decision Support System [DSS] Pharmacy Data, and the National Prosthetics Patient Database).

Interviews

Overview There will be three rounds of staff/provider interviews conducted at baseline, at the end of active implementation, and at or near the end of the sustainability period (for WAVE 1 and 2 sites); this will include interviews to inform the Business Case from hospital administrators and/or those in the financial office.

Baseline interviews (in-person or virtual) Dr. Sico and one other member of the research team will conduct on-site, in-person or virtual (dependent on travel restrictions in place due to COVID 19) baseline site visits, during which time individual semi-structured interviews will be conducted with champions and staff/providers who are involved in stroke/TIA care and will include: members of the stroke team (usually neurology and nursing), sleep medicine, the hospitalist service, primary care, and systems redesign. Interviews will address the planned approach for ASAP implementation (e.g., unattended PSG), local context related to stroke/TIA patient care, local champions, plans to coordinate the care of Veterans across services, and how best to incorporate systems redesign personnel throughout the intervention and sustainability phases.

Post-Active Implementation Interviews (telephone or virtual) Follow-up semi-structured interviews will be collected by telephone or by approved VA virtual platform (e.g., Microsoft Teams, etc.) in the month before the active implementation period ends and again at the end of sustainability. Interviews conducted at the end of the active implementation period will be conducted among champions and staff/providers who participated with ASAP and will seek to understand their experience with the ASAP program (including the intervention components and the implementation strategies) and seek to understand how ASAP will be sustained at their facility.

Sustainability Period Interviews (telephone or virtual) Interviews conducted after the sustainability period will be guided by the Dynamic Sustainability Framework and will be conducted among subjects at WAVE 1 and WAVE 2 sites.[30] Interviews will be conducted among ASAP end users regarding the intervention, contextual elements of implementation, and the VAMCs instituting

ASAP, with special attention to sustainability constructs of time, continued delivery, behavior change, evolution, and continued benefit. Examples of interview questions will include: What activities have you yourself participated in terms of implementing the local ASAP program? What would you say are the strengths of how the program has been implemented? What barriers have you and/or the team faced during making improvements in sleep apnea care? How did you overcome these barriers? How confident are you that ASAP will be sustained at your local VAMC? Why or why not? What local resources have been secured or provided for ASAP? What additional resources are still needed to sustain ASAP at your facility?

Veteran interviews (virtual) Dr. Sico and / or another member of the research team will conduct virtual, individual semi-structured interviews with Veterans who have participated in the ASAP intervention. Interviews will address the Veteran's experience with the ASAP intervention.

Surveys and Standardized Assessments

Overview Two surveys will be completed by staff/providers, the Organizational Readiness to Change Assessment (ORCA) [27,28] and the Program Sustainability Assessment Tool (PSAT) scores.[29] ORCA will be completed once; the PSAT will be completed three times for WAVES 1 & 2 sites and twice for WAVE 3. A standardized REDCap monthly site self-assessment.

ORCA (survey) VAMC employee subjects will also receive invitations to complete an online version of the ORCA.[27,28] The ORCA will be used to more thoroughly understand the readiness of each VAMC from the perspective of each PI across the primary scales of evidence, context, and facilitation and their subscales; of particular interest will include subscales of context (e.g., leadership practice and resources). The ORCA survey will be completed at baseline. Of note, elements of the ORCA will be incorporated into phone interviews conducted at the end of active implementation. Subjects will not complete a second ORCA survey.

PSAT (survey) VAMC employee subjects agreeing to participate will receive a link to complete the PSAT, an online tool which allows users to: (1) understand factors associated with sustainability; (2) assess the sustainability of a program; (3) review their sustainability report, and; (4) develop an action plan to enhance the chances of programmatic sustainability.[29] Eligible, participating staff/providers will receive invitations to complete the online instrument at three time points for WAVE 1 and 2 sites: at baseline, end of implementation, and end of sustainability and two time points for WAVE 3: at baseline and end of implementation. This tool assesses the facility's overall capacity for sustainability and across the specific domains of: environmental support, funding stability, partnerships, organizational capacity, communications, and strategic planning. Reports are generated by this tool, ranking the facility from 1 (little to no extent) to 7 (to a great extent) and provides guidance on what areas can be addressed to maximize sustainability.

REDCap Monthly Site Self-Assessment (standardized assessment) Will be completed by these subjects on a monthly basis during active implementation, and includes items related to triumphs, barriers, goals, adaptations to their QI

initiatives, and how COVID-19 and Philips Respironics Recall impacted ASAP implementation.

Assessments Completed by ASAP Research Team regarding ASAP Implementation

Overview Includes the Champion Measure of Performance (CHAMP) measure and (PRIUS) Prospectively-Reported Implementation Update and Score and are conducted by ASAP Research Team members.

CHAMP measure will be captured six times in total (once at baseline, three times during active implementation, once at the end of active implementation and once at the end of sustainability [however, only for WAVE 1 and 2 sites]). CHAMP measure will be completed by core research staff via a REDCap survey and the survey will be anonymous, confidential, and voluntary. As well-recognized components of developing, initiating, and implementing sustainable changes within healthcare systems, champions are adept at activating resources, advocating for innovation, and traversing socio-political environments within organizations.[82, 83] Champions will be intimately involved in implementation, meeting monthly with the ASAP team and systems redesign personnel to assess progress and barriers to implementation and to set and modify programmatic goals. We will encourage and support the champions as they embrace their role as change agents and help them develop their skills in champion-related activities: leading a team, advocating for ASAP within their work environment, motivating staff, making a business case to leadership, reflecting on and evaluation of diagnostic, treatment, recurrent vascular event data and building relationships with key stakeholders. Champions are important agents of change who promote the uptake of evidence-based practices within healthcare settings. We will collect the CHAMP measure to capture the perspective of those who the champion will "lead" into and through the ASAP project and by core research staff. The CHAMP measure will be captured six times in total (once at baseline, three times during active implementation, once at the end of active implementation and once at the end of sustainability [however, only for WAVE 1 and 2 sites]). ASAP will also develop the lean/systems redesign skills of local champions; supporting local champions in seeking yellow- or green-belt certification. These activities will take place during the kick-off, monthly collaborative conferences, and one-on-one coaching. Of note, the CHAMP measure was included as part of the initial cIRB approval packet as an appendix at the end of the baseline interview guide. Given the length of the interview guide, we opted to move this to a REDCap survey. Rather than asking the questions during the semi-structured interview we are having core ASAP Research staff complete these surveys.

PRIUS VAMC employee subjects agreeing to participate will receive monthly calls via telephone or Teams from research staff. Responding to the prompts, "What are some things that happened over the past month that seem relevant from your perspective to the implementation of this project?" Individuals score each update with a number ranging from +3 to -3. The PRIUS has shown to be

an efficient, structured method for developing a granular and context-sensitive account of the implementation progress.

GO Score During active implementation, the GO score will be determined using a real-time secret ballot among ASAP Research personnel via PollEv.com during Implementation Core meetings for each site at baseline (i.e., soon after the site kick-off) and then every 6-7 months during active implementation (which will align with the 7-month data periods in active implementation), at the end of active implementation and at the end of sustainability for WAVES 1 and 2 (Figure 2).

Category		GO Score	Description
ADVANCED		10	Nationally Recognized for Excellence in Providing ASAP Care
		9	ASAP System Plus Real-Time Monitoring
INTERMEDIATE	ASAP System of Care, Stage I	8	ASAP System of Care
	Comprehensive Program, Stage II	7	ASAP Practices Defined by Facility-Wide ASAP Program: 24/7
BASIC	Comprehensive Program, Stage I	6	ASAP Practices Defined by Facility-Wide ASAP Program: Regular Business Hours
	Discrete Components and Projects	5	Implementation of Facility-Wide Approach Specific to ASAP
		4	ASAP Practices Influenced by Cross-Service Processes, with Development of Facility-Wide Approach Specific to ASAP
	Discrete People	3	ASAP Practices Dominated by Organizational Silos and Individuals, with Some Collaboration Around Specific ASAP practices
		2	ASAP Practices Defined by Organizational Silos and Individuals, with Some Individual-Driven ASAP Activity
		1	ASAP Practices Defined by Organizational Silos and Individuals.

Figure 2. Group Organization Score for Implementing an Acute Sleep Service for Ischemic Stroke and Transient Ischemic Attack Patients in Addressing Sleep Apnea Post-Stroke/TIA (ASAP)

Consolidated Framework for Implementation Research

The ASAP Research team will directly apply implementation science constructs to the qualitative data. Using methods developed by Dr. Miech for RE-INSPIRE to rate and score for construct and valence (i.e., positive, neutral or negative)[42,43] across the domains of Process (Planning, Reflecting and Evaluating, and Champions), Inner Setting (Goals and Feedback, Networks and Communications, and Access to Knowledge & Information), Outer Setting (External Policy and Incentives), and Interventional Characteristics (Evidence Strength & Quality).

Notes from Interactions the ASAP Research Team has with Champions and Others Involved in Local Implementation of QI ASAP Initiatives

External Facilitation interactions between ASAP Research personnel and local site personnel performing ASAP QI will include on average a monthly scheduled meeting as well as *ad hoc* interactions that occurs at the request of either local teams or ASAP Research personnel. Number of minutes by participant by type of contact will be recorded in an external facilitation tracker and notes from these interactions will be recorded in on a standardized external facilitation interaction template

Monthly Collaborative Calls will produce a combination of data which includes triumphs and barriers, lessons learned from the prior month, and short- and longer-term implementation goals reported by sites, notes taken by ASAP Research personnel, and an ASAP Research-personnel debrief that is recorded and transcribed after ASAP Collaborative calls

Chart review from ASAP patients and patients at control sites

Charts will be reviewed by ASAP Field Staff using local CPRS and centrally using Compensation and Pension Records Interchange (CAPRI) access to the electronic health records, by trained staff at the Indianapolis site using methods successfully employed in other quality assessment projects.[86-88]

Administrative Data

Sources will include Corporate Data Warehouse [CDW] data, VA vital status files, VHA Support Service Center [VSSC], Decision Support System [DSS] Pharmacy Data, and the National Prosthetics Patient Database). These will include ICD codes for

5.7 Data Analysis

5.7.a. Data Analysis Overview A stepped-wedge trial will be used, rather than an individual patient-level randomized trial, because the intervention program is implemented at the site-level. Although this trial could have been set-up as a parallel cluster randomized trial (CRT), we sought to examine the treatment effect of the ASAP program using fewer sites (given the resource constraints of the IIR program). Additionally, we chose the stepped-wedge design, as opposed to a before and after within each cluster design, because the latter approach may be biased, (e.g., if underlying rates of OSA diagnosis change during the study period). With a stepped-wedge we can account for the time-related trends by incorporating time as a covariate. Time may be included in the analysis as a categorical variable (i.e., allowing a time effect at each step), or using time as continuous variable (assuming a linear increase or decrease over time).

5.7.b. Period Classification The stepped-wedge design allows for 8 mutually exclusive, 7-month data periods. Effectiveness outcome data for each site for each data period will be classified as either control or intervention. The active implementation period will be considered as the intervention phase. If a patient has more than one stroke/TIA, then only the first will be included in the analysis (i.e., patients in each time increment are unique). If a patient has more than one stroke/TIA event, but one occurs in the baseline period and one during the active implementation period, than the event during the active implementation period will be used. The stepped-wedge design allows site-level estimates of

proportions on 8 different cross-sectional samples. Thus, we will have repeated information on each site.

5.7.c. Specific Aim 1 (Effectiveness) Analyses Program effectiveness will be evaluated by a single, primary outcome (diagnostic rate). The diagnostic rate is defined as PSG completion within 30 days of presentation for the index stroke or TIA. **Effectiveness Outcome Ascertainment** We will seek to iteratively improve the CDW algorithms by comparing data to that of the chart review data (e.g., NIHSS data). COVID-19 data has been added to our ASAP administrative data set. COVID-19 data will consist of a combination of: (1) patients with polymerase chain reaction or antigen test results positive for severe acute respiratory syndrome coronavirus 2 (SARS-CoV-2) and; (2) from the VA National Surveillance Tool (NST). Also, pertinent dates related to the impact of the Philips Respironics Recall, indicating VHA-wide availability of PAP, have been added to our data.

Admissions for ischemic stroke and TIA to VAMCs have shown a decline during the COVID-19 pandemic. Furthermore, patients admitted to a VAMC with COVID-19 are not candidates to receive positive airway pressure (PAP) during the index hospitalization.

In considering analyses of the primary outcome, a sensitivity analysis will include re-running the final model with the inclusion of a facility-level, time-varying measure of COVID-19 burden. Although, inclusion of control sites which allows us to adjust for secular time trends may be sufficient to account for the effect of COVID-19 in the model.

We would also merge these COVID-19 data with data from an operational/quality improvement survey we conducted with Dr. Claibe Yarbrough to understand how COVID-19 affected the services provided by the Sleep Program across VHA, as well as to understand how VAMC sleep programs might move forward once a reopening occurs. Analyses using the COVID data would include whether a higher burden of COVID-19 within a responding facility was associated with higher rates of self-reported interruption of sleep services offered within a facility. We will obtain Medicare data for all stroke/TIA patients during the active implementation period; however, given delays in Medicare data availability, we will only plan to include these data in the primary analysis if they are available for all 3 time periods. We anticipate having no more than 1 million subjects within our administrative data sets. This number has been approximated with the inclusion of the COVID population, which at this time, continues to grow. Secondary to the large scale of the COVID population, we have been generous in our estimation of those included in the administrative cohort.

Control Sites

We will identify usual care control sites to examine temporal trends in outcomes among non-intervention sites. Our plan is to identify 5 to 6 controls for every participating ASAP site, if that is not possible (due to matching constraints), then we will plan to use 4 controls for each ASAP site. For example, we will use administrative data to examine the use of polysomnography across stroke/TIA patients in the VA system and compare changes in matched controls versus our intervention sites on the diagnostic rate. We will employ the same adjustment approach as described above for control sites.

Control sites will be matched to intervention sites on the basis of:

- Cerner status (all intervention sites are using Vista/CPRS)
- Volume of TIA and ischemic stroke patients
- VA Complexity level (all of the intervention sites are Level I; therefore, all control sites will also be Level 1)
- Baseline diagnostic rate

Given that some VAMCs do not provide acute stroke care, we will examine the proportion of stroke/TIA patients who were transferred out of the facility within 2 days of presentation as a measure of how well the sites are matched. If there is imbalance in this measure, we may add it as an explicit matching criterion.

Update: Eligible control sites were restricted to be Level 1 complexity and using Vista/CPRS similar to intervention sites. Sites were then matched on volume of TIA and ischemic stroke patients and baseline diagnostic rate. The transfer rate was compared and was less than 3.5% at all control and intervention sites thus was not used as a matching criterion.

Table 3. Diagnostic Rate		Active vs Control	Sust. vs Control	Sust. Effect	Active	Sust. Effect	Sust. vs Control	Active vs Control	Sust. vs Control	Sust. Effect
		$H_0: \theta = 0$	$H_0: \Upsilon = 0$	$H_0: \Upsilon = \theta_M$	$H_0: \theta = 0$	$H_0: \Upsilon = 0$	$H_0: \Upsilon = \theta_M$	$H_0: \theta = 0$	$H_0: \Upsilon = 0$	$H_0: \Upsilon = \theta_M$
Control	Active	CV = .3			CV = .5			CV = .8		
7%	13%	0.77	0.14	0.27	0.74	0.13	0.26	0.72	0.12	0.25
7%	13.50%	0.84	0.15	0.31	0.81	0.14	0.29	0.79	0.13	0.28
7%	14%	0.89	0.17	0.35	0.86	0.16	0.33	0.85	0.15	0.32
7%	15%	0.95	0.21	0.44	0.93	0.19	0.41	0.93	0.18	0.4

Power and Sample Size Analysis We will use randomization to determine the order in which the 6 participating intervention sites will receive the ASAP program.[89-94] Our primary power analysis is driven by the stepped-wedge design using large-sample theory[31,32,33] that site-level sampling proportions are

distributed approximately normal. Based on this assumption, we estimated the statistical power to detect the treatment effect β, while varying the intra-cluster correlation.

Diagnostic Rate

(Power for Diagnostic Rate: Active Implementation versus Baseline)

Using a stepped-wedge with 6 VA sites, 8 total time points, 2 sites switching at each step, and approximately 64 patients per site (111 x 7/12) at each step, we should have over 90% power to detect a difference in diagnostic rate of 7% and 15% while varying the coefficient of variation (or intra-cluster correlation). <u>7% was the observed diagnostic rate in FY2017, and intervention is expected to increase the diagnostic rate to 70% so we are appropriately powered for this primary outcome</u>. The detectable intervention diagnostic rate is provided in the table based on the two-sided test statistic while varying the coefficient of variation.[33] We additionally ran 1,000 simulations with varying treatment effects, including a large random site-effect $u_j \sim N(0, \tau^2)$, with τ^2 based on ICC (.03, .05, .20), a linear time-effect ($\gamma = 0.005$), and assuming a diagnostic rate in the control phase of 7%. We then fit the proposed linear mixed model with time as a categorical variable. Even in the extreme case that our ICC is large (ICC = 0.2), we are still adequately powered to detect the difference in diagnostic rate of 7% and 15% (Table 3). This initial power calculation was revisited to include additional baseline data and a slight reduced sample size because of the COVID-19 pandemic. The results of the updated power calculation did not differ substantively from the initial (above) power calculation.

Secondary Analyses Effectiveness The three secondary effectiveness outcomes are 30-day treatment rate, 90-day recurrent vascular event rate and 90-day all-cause readmission rate.

30-day Treatment Rate is defined as PAP treatment initiation within 30 days of presentation to the facility. The mean facility 30-day treatment rate is defined as PAP initiation within 30 days of admission. For this outcome, the denominator will be patients who are newly diagnosed with OSA. Patients with a prior diagnosis of sleep apnea (regardless of CPAP usage) and those who die within 7 days of discharge will be excluded. Patients who have a new diagnosis of central sleep apnea or Cheyne stokes (within 30-days of admission) or who are using Adaptive Servo Ventilation (ASV) are excluded. There may be no (or very few) patients in the baseline phase with OSA who are given treatment. Therefore, we will collapse data across all control time increments and all intervention time increments for each site.

90-day Recurrent Vascular Event Rate includes an ED or hospital admission for any of the following: TIA, stroke, myocardial infarction (MI), acute coronary syndrome (ACS), arrythmia, congestive heart failure (CHF), and all-cause mortality. The recurrent vascular event rate is measured from the day discharge.

Patients who are transferred out from the index stay are excluded. We expect the intervention only to reduce recurrent vascular events among patients with OSA who receive and adhere to treatment.

90-day All-Cause Readmission Rate includes an inpatient admission for any cause at either a VA or non-VA acute care facility. Planned readmissions are not included in the numerator. Patients who are transferred out from the index stay are excluded from the denominator. Exclude patients who were transferred to an acute care facility at any time during the index admission. As with the recurrent vascular event rate, we expect the intervention only to reduce readmission among patients who are diagnosed with OSA who receive and adhere to treatment.

Baseline Characteristics We expect that the characteristics of the stroke/TIA patients will remain relatively constant at each site across the different time points. We will summarize patient characteristics (e.g., demographics) by site and time. Any differences will be considered when interpreting results of analyses.

Analysis of the Primary Effectiveness Outcome

Diagnostic Rate Analysis

A generalized linear mixed model will be fit to the patient-level outcome of diagnostic testing (yes/no) to conduct facility-level inference. The primary effect of interest is the effect of the intervention during active implementation versus baseline. Control sites are included to add accuracy in estimation of the secular time trend. A non-exact match approach is planned for this analysis (i.e., unconditional) since this model is being fit to patient-level data which will be used to conduct facility-level inference and there is no guarantee that patients in control sites that were loosely matched to an intervention site using facility factors are similar. This approach will use all available control site data without pairing specific control sites to each ASAP site. Effects in the model include a site-level indicator of intervention site vs control site, indicator for each site and data period corresponding to active implementation or sustainability phase, and time (as categorical, indicating each 7-month data period). The model will also include a random intercept for site to account for potential correlation among patients from the same site.[36] Our interest is in testing the effect of the ASAP intervention on the proportion of patients who are diagnosed at the site-level. This model will also be fit adjusting for any important patient covariates (i.e., stroke severity[37]). Both unadjusted and adjusted results will be reported. Contrasts from the model will also be estimated. All statistical tests will be two-sided, with p-values < 0.05 considered statistically significant. All analyses will include checking of assumptions and model fit.

Analysis of Secondary Outcomes As described above, because the <u>treatment</u> rate can only be assessed among the patients who have been diagnosed, we will be limited in our analysis of treatment. If several patients are diagnosed in the during the control phase (which is unlikely), we would use the same mixed model approach as with other outcomes. However, because we expect to have very

sparse data for the baseline phase, we plan to collapse data within each site across the baseline phases and the treatment phase and compare the within site treatment rates using a permutation test. Doing so will randomly flip the treatment labels of site-level treatment rates. Because there are 6 sites, there are 2^6 possible permutations of the treatment labels within pairs. The p-value will then be the proportion of permutations that lead to a test statistic at least as extreme as the actual data. Additionally, we will report the estimated site-level treatment rate during the baseline and intervention phases as well as the associated 95% CIs. We will also compare the overall treatment rate among all patients in the baseline phase to all patients in the active implementation phase using Fisher's exact test.

90-day Recurrent Vascular Event Rate and Readmission Analysis will be analyzed similarly to the primary outcome with the exception that the 7-month code-red data period (beginning 9/2021) will not be included. Models for the secondary outcomes (excluding treatment rate) will be fit without covariates (unadjusted) and additionally including patient-level factors (adjusted).

Missing Data Because we are using centralized chart review augmented by data from the CDW to collect our baseline characteristics and outcome data, we expect to have very little missing data. Only a few of the patient-level variables which may be included in the models adjusting for covariates may contain a small amount of missing such as stroke severity (as measured by the NIHSS). The amount of missing will be examined before deciding on the handling of missing data. Either complete-case or multiple-imputation will be used for models adjusting for patient-level factors. In the highly unlikely event that for a certain time period we are not able to get data from a specific site, the mixed effects model allows us to still use data for the other time periods for that site. The proposed mixed model methods are appropriate as long as data are missing completely at random (MCAR) or missing at random (MAR). If for any reason, we suspect the pattern of missing data is non-ignorable, more complex modeling approaches may be used.[40,41]

Specific Aim 1 Sensitivity Analyses Given that implementation of ASAP may require some time after the kick-off we will examine intervention effectiveness if the intervention is considered starting in the second 7 months of the active implementation period. To do this, we will use the same generalized linear mixed model approach with the only difference being that sites will not be considered as being in the intervention phase until the second 7 months of the active implementation phase. A sensitivity analysis will also be conducted for the primary and secondary outcomes (except treatment rate) by including a time-varying, facility-level covariate for COVID-19 burden. Also, due to the Philips Respironics PAP machine recall, the national VA PAP recall level (green, yellow, red) was also collected. We plan to include all data periods for diagnostic testing for the primary analysis and remove the 7-month data period during which time the VA was in red status from analysis for all other outcomes, since theoretically patients could still be diagnosed even if treatment cannot be provided. However, since some sites did not even diagnose during the status red time, due to the ethical concerns of not being able to treat if the patient was found to have sleep

apnea, a sensitivity analysis will be examined where the 7-month code red period is removed also for the primary outcome. If feasible, we may explore the use of all data by including time-varying variables for VA PAP recall level (green, yellow, red) if the time-varying variable can be properly constructed for the 7-month time periods.

5.7.d. Specific Aim 2 (Implementation) Analysis

Interview Administration and Analysis of Implementation Data

Interviews (with staff/providers, and Veterans) will address the planned approach for ASAP implementation (e.g., unattended PSG), local context related to stroke/TIA patient care, local champions, plans to coordinate the care of Veterans across services, and how best to incorporate systems redesign personnel throughout the intervention and sustainability phases (at WAVE 1 and 2 sites). Participating staff/providers will also receive invitations to complete an online version of the Organizational Readiness for Change Assessment (ORCA).[27,28] The ORCA will be used to more thoroughly understand the readiness of each VAMC from the perspective of each PI across the primary scales of evidence, context, and facilitation and their subscales; of particular interest will include subscales of context (e.g., leadership practice and resources). Follow-up semi-structured interviews will be collected by telephone or virtually immediately after the active implementation period and again at the end of sustainability. Elements from ORCA facilitation subscales will be investigated at the follow-up period (e.g., senior leadership management characteristics, resources) as well as the aforementioned context subscales. These interviews will be audio-taped, transcribed verbatim, and reviewed by investigators to ensure accuracy of transcription. We will develop detailed case studies and timelines to integrate and analyze data related to each site's specific approach to developing and refining a site's acute sleep apnea testing and treatment program. Transcripts, case studies, and timelines will be imported into the software program NVivo, which will be used to support the qualitative data analysis, including coding, analysis, and memo-writing.

Throughout the qualitative data analysis process, investigators will write analytic memos to document the evolution of the analyses, the emergence of early patterns and possible findings, the convergence of major themes, the challenges posed by the analysis, and ideas related to hypothesis testing; these analytic memos will form the basis for conclusions and findings. Investigators will explore disconfirming evidence and consider possible rival explanations that contrast with study findings and conclusions. Investigators will share preliminary findings with each other on a weekly basis to solicit feedback, including observations and questions.

We will evaluate the intervention components of the system redesign Virtual Collaborative and the data monitoring as well as the implementation strategies at the end of the active implementation stage and again at the end of sustainability. Data obtained during active implementation at earlier sites will be used to modify the ASAP program for subsequent waves, whereas data obtained during the

sustainability period will be used to understand factorsassociated with sustainable implementation of the ASAP program. Qualitative analyses will involve two qualitative researchers independently reading and re-reading the data, assigning labels and codes to data segments, and developing initial themes from the data. The analysis team will meet to develop consensus on initial themes and related codes from the data, with revision to the themes and codes using an iterative process.

Categorical data will be captured by the ASAP research team will include individual-level data (e.g., job position, roles assumed and activities conducted in support of ASAP implementation). We will use NVivo to integrate the tagged qualitative data, the scored construct data, the standardized ORCA data, the categorical data, and the quantitative outcome data in a single, unified project file with multiple codebooks (qualitative, scored constructs, quantitative, categorical). We will use the NVivo "matrix query" feature to explore relationships between the qualitative, construct, quantitative and categorical data, and through this direct cross-referencing of qualitative and quantitative data iteratively analyze how specific combinations of intervention components, implementation strategies and features of local context influence project outcomes. We will also collect data regarding implementation activities proposed and conducted by the sites during the kick-off and throughout active implementation to understand the activities they undertook to conduct quality improvement initiatives related to ASAP. Implementation activities will also be categorized based on their frequency, category (e.g., electronic health record-based), and relative complexity. ASAP analyses will focus both within and across facilities, with special attention to facility- and patient-level factors contributing to increased uptake of the ASAP protocol.

CFIR and GO Score Ratings After each 7-month data period, the ASAP research team will meet to conduct CFIR scoring, taking into account memos and information gleaned from data sources outlined in Section 5.6. to score each VAMC with a facility-level valence (i.e., positive, neutral, negative or minimally referenced) and magnitude (i.e., weak or strong) for each CFIR construct.[42-44] These data sources will also be used to inform GO Score ratings.

As data will be collected longitudinally across, the study team will also identify trends in CFIR and GO Score as they change over time while using memos from such sources as PRIUS calls and External Facilitation calls to understand reasons for change across 7-month data periods. ASAP findings should thus be well-positioned to contribute not only to implementation science but also to the VHA itself as part of a learning healthcare system.

Case Comparisons We will use the constant comparison technique to identify emerging themes in the data, including special attention to themes related to context, champions, and external facilitation. Dependent on variability seen between contextual elements, CFIR construct scoring and GO Scores over time, comparisons will be made on higher versus lower performing sites.

Veteran interviews will be conducted at each site to gain the Veteran's perspective on the implementation and their personal experiences as the recipients of local ASAP initiatives.

Configurational Analysis draw on concepts from set theory and Boolean algebra to identify conditions that make a difference in reaching an outcome of interest among a defined set of cases.(36, 37) Configurational Comparative Methods (CCMs) offer a set of rigorous, mathematical methods to examine multifactorial causality (i.e., when several variables together have a joint effect on an outcome) as well as equifinality (i.e., several distinct paths leading to the same outcome). CCMs focus on cases, conditions, and combinations rather than variables, and answers research questions like "What combinations of conditions were difference-makers to the outcome of interest?" Configurational approaches have increasingly been used in implementation science and health services research.(21, 38, 39) Within the broader family of CCM approaches, this study applied multi-value Coincidence Analysis (CNA), which offers a systematic approach to build redundancy-free models with small sample sizes and to assess equifinal pathways to implementation success.(21) Factors that will be considered in the configurational analysis will be calibrated after data collection was complete. To develop the analytic dataset for the configurational analysis, we drew on factors collected during the active implementation period of the ASAP trial. The calibration approach is described in Table 1. Whereas some factors are dichotomous (presence/absence, continuous data may be calibrated into quartiles or tertiles.

5.7.e. Specific Aim 3 (Sustainability Analysis)

Survey and Interview Administration and Analysis of Implementation Data
Those agreeing to participate will receive a REDCap invitation to complete the PSAT. PSAT allows users to: (1) understand factors associated with sustainability; (2) assess the sustainability of a program; (3) review their sustainability report, and; (4) develop an action plan to enhance the chances of programmatic sustainability.[29] Participating staff at WAVE 1 and 2 sites will receive invitations to complete the online instrument at three time points: at baseline, at the end of active implementation, and at the end of sustainability. WAVE 3 sites will complete the PSAT at baseline and at the end of implementation. This tool assesses the facility's overall capacity for sustainability and across the specific domains of: environmental support, funding stability, partnerships, organizational capacity, communications, and strategic planning. Reports are generated by this tool, ranking the facility from 1 (little to no extent) to 7 (to a great extent) and provides guidance on what areas can be addressed to maximize sustainability. Of note, as reflected in our letters of support from our site co-investigators, they have obtained support and commitment from their institutions and did not score below a 5.0 on their PSAT report. Telephone or virtual interviews will be conducted in after the final PSAT has been completed

and at the end of sustainability for sites within WAVE 1 and WAVE 2. We intend to conduct phone or virtual interviews among seven ASAP end users at each of the six sites. Guided by the Dynamic Sustainability Framework[30], we will both conduct interviews among ASAP end users regarding the intervention, contextual elements of implementation, and the VAMCs instituting ASAP, with special attention to sustainability constructs of time, continued delivery, behavior change, evolution, and continued benefit. Examples of interview questions will include: What activities have you yourself participated in terms of implementing the local ASAP program? What would you say are the strengths of how the program has been implemented? What barriers have you and/or the team faced during making improvements in sleep apnea care? How did you overcome these barriers? How confident are you that ASAP will be sustained at your local VAMC? Why or why not? What local resources have been secured or provided for ASAP? What additional resources are still needed to sustain ASAP at your facility? PSAT data will be added to the NVivo project files containing the tagged qualitative interview data, the scored construct data, the standardized ORCA data, the categorical data, and the quantitative outcome data, and analyzed to understand barriers and predictors of sustained use of ASAP and to understand how intervention components, implementation strategies and local context influence sustainability. We hypothesize that sustainability will be greatest at sites that use HST as their primary approach and have champions who engage with systems redesign staff and perform at least quarterly implementation activities. To estimate if the diagnostic rate continues through the sustainability phase, the effect for sustainability will be estimated from the primary outcome models.

5.7.3. Specific Aim 4 (Business-Case Analysis)

Business-Case Analysis Overview We will construct a business-case analysis that involves a comparison of the ASAP program with the current standard practice. If staff time data collected from the sites is adequate, we will construct a business-case analysis at each of the participating sites as well as an aggregated (overall) business-case analysis. We will meet with the facility leadership for participating sites at the beginning of the study and confirm that the proposed business-case analysis includes all of the key financial elements that are of interest to the facility leadership. We will also ask whether there are important non-financial considerations that would inform a decision about whether to invest in the ASAP program. For example, for some interventions, key non-financial aspects can be so important to facility leaders that they need to be considered in a business-case analysis (e.g., improved clinic access). We do not anticipate that the ASAP program will involve elements not able to be translated into financial terms, and hence our primary plan is to focus on financial elements only. Involving facility leadership in the development of the business-case analysis serves two key functions: it ensures that the analysis includes elements that may not have been apparent to the research team members and it strengthens buy-in for the program with the key stakeholders. This latter issue is of relevance to implementation

projects where the goal is to ensure strong leadership support for program implementation and sustainability like ASAP.

Table 5. Data Elements and Sources Related to Business-case Analysis	
**Data Element**	**Data Sources**
Staff time (training)	Locality-specific staff pay rates will be obtained from Financial Management System (FMS), MCA, and Account-Level Budgeter Cost Center (ALBCC) databases; Staff participants will maintain a diary of time spent in training—an average time will be used for the main analysis and extreme values will be used in sensitivity analyses
Staff time (implementation)	Surveys of participants involved in ASAP implementation
Equipment: PSG-related	Will include any one-time costs (e.g., PSG equipment purchases) and also incremental per-patient costs associated with disposable items (e.g., gloves, tape). Costs will be obtained from prices that are paid by the sites and as above the average cost will be used for the main analysis. and extreme values will be used in sensitivity analyses.
Total healthcare costs for one-year pre- and post-index event	Total healthcare costs (inpatient and outpatient) for the individual patients will be obtained from MCA data. All costs will be converted to the same fiscal year (to adjust for temporal differences in the waves).

The business-case analysis will focus on financial costs and savings. We will include all of the ASAP program costs (Table 5) including: staffing and training costs (obtained by staff surveys about time allocations for specific activities which will be converted to staffing costs), equipment costs (obtained by sleep medicine service surveys), and supply costs (obtained by site PI surveys); patient travel reimbursements (if patients are traveling to the VA to obtain sleep services; obtained from VSSC Beneficiary Travel report[45]); and total patient health care costs for inpatient and outpatient utilization (obtained from the VHA Managerial Cost Accounting [MCA] System). We do not expect that the ASAP program will involve changes in space, but if any of the participating sites requires changes in space this will be included in the analysis. We will not include any research-specific costs. The business-case analysis will be built from the VAMC leadership perspective and will therefore not include patient costs. The timeframe for the business-case analysis will be the active implementation phase and no discounting will be performed. If data availability allows, we will compare the business-case analyses across the 6 participating sites. The primary comparison will involve assessing differences between intervention patients versus usual care patients. Veterans Equitable Resource Allocation (VERA) reimbursements will not be considered in the primary business-case analysis because all the patients in the ASAP program have had a stroke or TIA and the intervention is unlikely to influence VERA classification or reimbursements, although we will ask site leadership regarding including VERA in the analysis. The final product of the business-case analysis will be a report and will include the estimated per patient cost for ASAP program implementation and differences in the estimated costs for intervention versus usual care patients. Cost data will only involve data from ASAP sites. As we anticipate that the ASAP program will reduce recurrent vascular events, we hypothesize that the business-case analysis will favor the intervention at sites with the highest baseline recurrent vascular event rate, reducing costs associated with relatively expensive readmissions.

Specific Aim 4 (Business-Case) Analysis:

For the business-case analysis, we will evaluate the effect of the intervention on annual patient-level healthcare costs after index admission, while adjusting for annual patient-level healthcare costs prior to admission. To do this, we will include 7-month data periods that occur during baseline and active implementation. Costs will first be converted to the same fiscal year to account for inflation using the Consumer Price Index (CPI). The basic stepped wedge model will be fit using a generalized linear mixed model approach assuming the costs follow a gamma distribution with log link. Model results will be evaluated to check assumptions. The model will include the prior annual healthcare costs as a covariate, time period (as categorical), and active implementation indicator. Additionally, a random site-level intercept will be included to account for any heterogeneity in costs due to unmeasured site-level factors. Some patients may die within 12 months of the index admission and other patients in the last wave of sites may have fewer than 12 months of cost data available due to administrative cutoff of the database. This information will be examined descriptively. Initial models may only use patients with complete cost information available. Alternatively, we will consider treating costs as right censored for those with incomplete information.

5.7 Withdrawal of Subjects

The patients do not actively enroll or withdraw from the study. If they meet the exclusion criteria, their data will not be included in the analyses.

Health care providers and administrators who are invited to participate in the study will always have the right to withdraw from the study at any time without repercussions.

4. Reporting

Assessment of level of risk: minimal

Oversight for this investigation will be provided by: Drs. Jason Sico (PI) and Dawn Bravata (PI)

Internal Data Monitoring Committee (DMC): Will be comprised of Drs. Sico and Bravata and the quantitative core (Drs. Laura Myers and Joanne Daggy). This DMC will meet at least quarterly and more regularly should concerns arise regarding data integrity. Note: reporting to the VA HSRD DMC will occur as required by that committee (anticipated to be annually).

Monitoring of Adverse Events and Safety Plan: Participants (specifically VHA Employees) will be monitored regularly for adverse events by the Program Coordinator. Staff members are trained to report any adverse events (AEs) promptly to the PIs. Participants will be encouraged to report any adverse events they have experienced. We anticipate that using this active identification method will allow us to identify all adverse events in a timely manner. Based on our prior experience with implementation trials, we do not anticipate that any participant will experience adverse events related to participation other than the expected inconvenience related to scheduling time for interviews, Kick-Off, and quality

improvement meetings. The Program Coordinators will be responsible for reviewing the adverse events reporting of all participants each day and reporting the information to the PIs.

Reporting of Unanticipated Problems or Adverse Events: As per VHA rules, we will notify the IRB promptly using the appropriate form when any serious adverse event occurs. If the incident is serious unanticipated or requires revision of the Project Description we will notify the IRB by telephone as soon as possible and always within 24 hours. A formal report will be provided within 2 business days. All adverse events will be reported yearly to the IRB for review regardless of seriousness or relationship to the research. Because of this policy, the IRB will be providing parallel review of adverse events along with the PIs. We believe this will insure stringent oversight and early identification of any unexpected risks to human subjects.

Accuracy and Integrity of the Data: Will be ensured in three ways. Firstly, the VA Connecticut Healthcare System and Richard L. Roudebush VAMC Centers for Health Services Research conduct computer operations in its secure Centralized Computing Facility managed by VHA OI&T network and server administrators. In each Center's computing facility, all research data are stored on VHA-Administered servers which are physically secured in either West Haven, CT, or Indianapolis, IN, VAMC computer rooms. VHA network access to research data is controlled in accordance with the Center's Standard Operating Procedures and VA policies and in cooperation with the VA Connecticut Healthcare System and Richard L. Roudebush VAMC IRBs. In addition, standard operating procedures have been developed to ensure servers, workstations and portable computers are kept up-to-date with virus filters, security patches, software updates and firmware updates. Secondly, the project coordinators will review a sample of the interviews to ensure adherence to the study protocol. Thirdly, a 10% random sample of all charts will be double abstracted to examine inter-rater variation. Variables with discrepancies will be reviewed by the whole chart review team to identify potential problems with the data collection tool or the data dictionary.

5. Privacy and Confidentiality

The ASAP program is an inherently low-risk project. The primary risks relate to the unauthorized release of study-related patient or staff information. Potential risk is characterized as not greater than minimal, that is, a degree of risk that is no greater than that encountered during daily activities. Participation in the study is not anticipated to be associated with any substantial discomfort or inconvenience. There is a small risk to subjects of loss of privacy or confidentiality.

All project staff members are professionals with extensive training related to required procedures for maintaining confidentiality and privacy. Additionally, specific efforts will be maintained to maximize the likelihood that research related information will be kept secure and unavailable to others not involved in the project. As described above, only a code number will identify research records. The code number will not be based on any information that could be used to identify a participant (for example, social security number, initials, birth date,

etc.). The master list will be kept separately from the research data and stored as an electronic data file behind the VA firewall. Only authorized persons will have access to the information gathered in this project. There is little risk to loss of privacy or confidentiality given these measures, and there has been no instance of a problem in this area in prior trials conducted by this investigative team.

In considering risks pertaining to confidentiality, the following safeguards will be in effect in ASAP:

1) Data Confidentiality – We have standard operating procedures for data acquisition and data management. These procedures were designed to protect against data loss and maintain patient confidentiality. These procedures have been developed and used in many studies and we will adhere to these procedures for the proposed study. Audio files and transcriptions related to interviews will be stored behind the VHA firewall on a secure VA-server. Computer files will be password protected. Files containing names or other personal identifiers will have a separate password and will be accessible only to personnel who need to contact subjects.

2) Breach of confidentiality – All participant information collected in the context of this research study, and even the fact that an individual is participating in the study, will be considered confidential. This confidentiality will be assured through several mechanisms. First, each participant will be assigned an anonymous study ID.. In addition, such materials, when in use, will be kept away from public scrutiny. Third, access to all participant data will be restricted to authorized personnel. In the case of computerized study data, access to data will be password protected, and staff members will be assigned individualized passwords that allow them access to only those elements of the data management system to which they are authorized. In addition, all study personnel will maintain certification with training in research ethics, which includes training on confidentiality. Finally, participants will not be identified by name in any reports or publications, nor will data be presented in such a way that the identity of individual participants can be inferred. All information obtained within the course of the study that identifies an individual will be treated as confidential in accordance with section 903C of the Public Health Service Act (42 U.S.C.299a-1). We will remove all identifiers from analytic data sets after data merging and keep all personal identifiers in a separate location from the analytic data. All research data files are organized by study ID number and have no names or other identification attached. This ID number links all computerized research records.

3) Data Security – We have developed standard operating procedures for data management in the conduct of previous trials. These procedures were designed to protect against data loss and maintain participant confidentiality. The study team will take steps to ensure the participant's confidentiality is protected at each step of the process. As stated above, participant will be assigned unique ID numbers (unrelated to their social security numbers or other identifiers). Our study data will be maintained on VHA network servers which are secured and

backed-up on a nightly basis. Access to these servers is controlled by network administrators.

4) Digital Audio Recording of VHA Employee and Veteran Interviews – Audio taping is necessary to ensure that all information during the interviews is collected. Participants who decline will not be eligible to participate in the interviews. Participants will be asked to provide verbal consent. They will also be asked to state their consent to audio recording of the interview at the beginning of the interview as soon is the recorder is turned on. The digital audio recording will be labeled with the subject's project ID# and will not contain any identifying information. Only approved research personnel will have access to the audio files, and they will be stored behind the VHA firewall.

6. Communication Plan

Dr. Sico, in conjunction with VA Connecticut Healthcare System and Richard L. Roudebush VAMC ASAP staff, will communicate with the Local Site Investigators (LSIs) and site-based ASAP staff regarding obtaining local site approvals. Staff within the two research coordinating centers will aid each site in completing requisite approvals. During the kick-off at the start of each wave for each site, ASAP-staff and the LSI will agree upon a schedule of meetings, which will include a scheduled meeting (likely once/month) and how best and when to convene ad hoc meetings. Calling an ad hoc meeting may be prompted based on the data monitoring where there is an unfavorable mismatch between the number of patients admitted who were eligible for the ASAP protocol and those who received it. For example, a site may have had 15 Veterans admitted who could have benefited from the ASAP protocol, whereas only 3 Veterans received it. In this example, the mismatch may be because of logistical concerns implementing the project or because the site where research is being conducted is not fully engaged. ASAP personnel will communicate about these concerns and, as necessary, perform a Plan-Do-Study-Act (PDSA) to determine how to improve implementation of the ASAP protocol. Should it be determined that a given site is not engaged in the project, Drs. Sico and Bravata will communicate with the Director to provide notification and to seek guidance on next steps.

In order to keep all 6 engaged intervention sites informed of changes to the protocol, informed consent, and/or HIPAA authorization, Drs. Sico and Bravata will send emails out to the LSI and appropriate local staff (e.g., site-based research assistants) regarding upcoming changes as well as the most updated forms. Should questions arise regarding updates, Dr. Sico has the contact information (including email addresses, VA-office phone number, and cell phone numbers) of each LSI. As such, an ad hoc meeting could be convened to discuss changes. Additionally, announcements regarding any changes will also be made on the Systems Redesign Virtual Collaborative/IOP. These same lines of communication will also be used to inform local sites of any serious adverse events, unanticipated problems, and/or interim results which may impact the conduct of the study. Sharing unanticipated problems as well as possible solutions is an important component of our Systems Redesign Virtual

Collaborative/IOP which will allow for sites to learn from one another and from interim study results.

In order to ensure that the study is conducted according to the IRB-approved protocol, ASAP study coordinators from VA Connecticut Healthcare System and Richard L. Roudebush VAMC will assist with reporting to VA Central IRB and each local VA research service regarding study progress. If a potential protocol violation has occurred, either as reported by an LSI and/or discerned during the course of chart review, Drs. Sico and Bravata will investigate the potential violation. If a violation has indeed occurred, Drs. Sico and Bravata will report the violation to VA Central IRB.

The facility directors and LSIs of the 6 intervention sites will be notified of the date of the kick off, which will be the start of active implementation for that site and the duration of active implementation for that site (Figure 1).

References

1. Williams L. VA Stroke Quality Enhancement Research Initiave (QUERI) Annual Report and Strategic Plan. In: Indianapolis, IN: VA Health Services Research and Development Service; 2010.
2. Kernan WN OB, Black HR, Bravata DM, Chimowitz MI, Ezekowitz MD, Fang MC, Fisher M, Furie KL, Heck DV, Johnston SC, Kasner SE, Kittner SJ, Mitchell PH, Rich MW, Richardson D, Schwamm LH, Wilson JA; American Heart Association Stroke Council, Council on Cardiovascular and Stroke Nursing, Council on Clinical Cardiology, and Council on Peripheral Vascular Disease. Guidelines for the prevention of stroke in patients with stroke and transient ischemic attack: a guideline for healthcare professionals from the American Heart Association/American Stroke Association. *Stroke*. 2014 45:2160-2236.
3. Meschia JF BC, Boden-Albala B, Braun LT, Bravata DM, Chaturvedi S, Creager MA, Eckel RH, Elkind MSV, Fornage M, Goldstein LB, Greenberg SM, Horvath SE, Iadecola C, Jauch EC, Moore WS, Wilson JA; on behalf of the American Heart Association Stroke Council, Council on Cardiovascular and Stroke Nursing, Council on Clinical Cardiology, Council on Functional Genomics and Translational Biology, and Council on Hypertension. . Guidelines for the primary prevention of stroke: a statement for healthcare professionals from the American Heart Association/American Stroke Association. *Stroke*. 2014 45:3754-3832. doi: 10.1161/STR.0000000000000046
4. Wessendorf T, Teschler H, Wang Y-M, Konietzko N, Thilmann A. Sleep-disordered breathing among patients with first-ever stroke. *J Neurol*. 2000;247:41-47.
5. Turkington P, Bamford J, Wanklyn P, Elliott M. Prevalence and Predictors of Upper Airway Obstruction in the First 24 Hours After Acute Stroke. *Stroke*. 2002;33:2037-2042.
6. Harbison J, Ford G, James O, Gibson G. Sleep-disordered breathing following acute stroke. *Q J Med*. 2002;95:741-747.
7. Iranzo A, Santamaria J, Berenguer J, Sanchez M, Chamorro A. Prevalence and clinical importance of sleep apnea in the first night after cerebral infarction. *Neurology*. 2002;58:911-916.
8. Dyken M, Somers V, Yamada T, Ren Z, Zimmerman M. Investigating the relationship between stroke and obstructive sleep apnea. *Stroke*. 1996;27:401-407.
9. Bassetti C, Aldrich M. Sleep Apnea in Acute Cerebrovascular Diseases: Final Report on 128 Patients. *Sleep* 1999;22:217-223.
10. Parra O, A A, Bechich S, García-Eroles L, Montserrat J, López J, Ballester E, Guerra J, Sopeña J. Time course of sleep-related breathing disorders in first-ever stroke or transient ischemic attack. *American Journal of Respiratory & Critical Care Medicine*. 2000;161:375-380.
11. Sandberg O, Franklin K, Bucht G, Gustafson Y. Sleep apnea, delirium, depressed mood, cognition, and ADL ability after stroke. *Journal of the American Geriatrics Society*. 2001;49:391-397.
12. Kapen S, Goldberg J, Wynter J. The Incidence and Severity of Obstructive Sleep Apnea in Ischemic Cerebrovascular Disease. *Neurology*. 1991;41:125.
13. Parra O, Sánchez-Armengol A, Bonnin M, Arboix A, Campos-Rodríguez F, Pérez-Ronchel J, Durán-Cantolla J, de la Torre G, González Marcos J, de la Peña M, et al. Early treatment of obstructive apnoea and stroke outcome: a randomised controlled trial. *Eur Respir J*. 2011 37:1128-1136.
14. Bravata D, Concato J, Fried T, Ranjbar N, Sadarangani T, McClain V, Struve F, Zygmunt L, Knight H, Lo A, et al. Continuous Positive Airway Pressure: Evaluation of a Novel Therapy for Patients with Acute Ischemic Stroke. *Sleep*. 2011;34:1271-1277.

15. Bravata D, Concato J, Fried T, Ranjbar N, Sadarangani T, McClain V, Struve F, Zygmunt L, Knight H, Lo A, et al. Auto-titrating continuous positive airway pressure for patients with acute transient ischemic attack: a randomized feasibility trial. *Stroke*. 2010 41:1464-1470.
16. Mohsenin V, Valor R. Sleep Apnea in Patients With Hemispheric Stroke. *Arch Phys Med Rehabil*. 1995;76:71-76.
17. Colten H, Abboud F, Block G, Boat T, Litt I, Mignot E, Miller R, Nieto J, Pack A, Parker K, et al. Sleep Disorders and Sleep Deprivation: An Unmet Public Health Problem In: Washington, DC: National Academy of Sciences; 2006.
18. Good D, Henkle J, Gelber D, Welsh J, Verhulst S. Sleep-disordered breathing and poor functional outcome after stroke. *Stroke*. 1996;27:252-259.
19. Cherkassky T, Oksenberg A, Froom P, Ring H. Sleep-related breathing disorders and rehabilitation outcome of stroke patients: a prospective study. *Am J Phys Med Rehabil*. 2003 82:452-455.
20. Turkington P, Allgar V, Bamford J, Wanklyn P, Elliott M. Effect of upper airway obstruction in acute stroke on functional outcome at 6 months. *Thorax*. 2004 59:367-371.
21. Kaneko Y, Hajek V, Zivanovic V, Raboud J, Bradley T. Relationship of sleep apnea to functional capacity and length of hospitalization following stroke. *Sleep*. 2003 26:293-297.
22. Loube D, Gay P, Strohl K, Pack A, White D, Collop N. Indications for positive airway pressure treatment of adult obstructive sleep apnea patients: a consensus statement. *Chest* 1999;115:863-866.
23. Balk E, Moorthy D, Obadan N, Patel K, Ip S, Chung M, Bannuru R, Kitsios G, Sen S, Iovin R, et al. Diagnosis and Treatment of Obstructive Sleep Apnea in Adults. In: *AHRQ Comparative Effectiveness Reviews*. Rockville (MD): Agency for Healthcare Research and Quality 2011
24. Giles T, Lasserson T, Smith B, White J, Wright J, Cates C. Continuous positive airways pressure for obstructive sleep apnoea in adults. *Cochrane Database Syst Rev*. 2006;3:CD001106.
25. Bravata D, Ferguson J, Miech E, Agarwal R, McClain V, Austin C, Struve F, Foresman B, Li X, Wang Z, et al. Diagnosis and treatment of sleep apnea in patients' homes: the rationale and methods of the “GoToSleep” randomized-controlled trial. *J Clin Sleep Med*. 2012;8:27-35.
26. VL P, S S. Continuous Positive Airway Pressure (CPAP). StatPearls Publishing. https://www.ncbi.nlm.nih.gov/books/NBK482178/. 2018. Accessed 5/6/2018.
27. Helfrich C, Yu-Fang L, Mohr D, Meterko M, Sales A. Assessing an organizational culture instrument based on the Competing Values Framework: Exploratory and confirmatory factor analyses. *Implement Sci*. 2007;2:13.
28. Helfrich H, Li Y, Sharp N, Sales A. Organizational readiness to change assessment (ORCA). *Implementation Science*. 2009;4:38.
29. Luke DA, Calhoun A, Robichaux CB, Elliott MB, Moreland-Russell S. The Program Sustainability Assessment Tool: A New Instrument for Public Health Programs. *Preventing Chronic Disease*. 2014;11:E12. doi: 10.5888/pcd11.130184
30. Chambers DA, Glasgow RE, Stange KC. The dynamic sustainability framework: addressing the paradox of sustainment amid ongoing change. *Implementation science : IS*. 2013;8:117-117. doi: 10.1186/1748-5908-8-117
31. Hayes R, Moulton L. *Cluster Randomized Trials*. Boca Raton, FL: Chapman& Hall/CRC; 2009.
32. Hussey MA, Hughes JP. Design and analysis of stepped wedge cluster randomized trials. *Contemp Clin Trials*. 2007;28:182-191. doi: 10.1016/j.cct.2006.05.007

33. Baio G, Copas A, Ambler G, Hargreaves J, Beard E, Omar RZ. Sample size calculation for a stepped wedge trial. *Trials*. 2015;16:354. doi: 10.1186/s13063-015-0840-9
34. Bravata DM, McClain V, Austin C, Ferguson J, Burrus N, Miech EJ, Matthias MS, Chumbler N, Ofner S, Foresman B, et al. Diagnosing and managing sleep apnea in patients with chronic cerebrovascular disease: a randomized trial of a home-based strategy. *Sleep & Breathing = Schlaf & Atmung*. 2017;21:713-725. doi: 10.1007/s11325-017-1494-5
35. Li J, Zhang Y, Myers LJ, Bravata DM. Power calculation in stepped-wedge cluster randomized trial with reduced intervention sustainability effect. *Journal of Biopharmaceutical Statistics*. 2019;29:663-674. doi: 10.1080/10543406.2019.1633658
36. Brown H, Prescott R. *Applied Mixed Models in Medicine*. New York: John Wiley & Sons; 1999.
37. Williams LS, Yilmaz EY, Lopez-Yunez AM. Retrospective assessment of initial stroke severity with the NIH Stroke Scale. *Stroke*. 2000;31:858-862.
38. Concato J, Peduzzi P, Holford T, Feinstein A. Importance of events per independent variable in proportional hazards analysis I: Background, goals, and general strategy. *J Clin Epideminol*. 1995:1495-1501.
39. Peduzzi P, Concato J, Feinstein A, Holford T. Importance of events per independent variable in proportional hazards regression analysis II: Accuracy and precision of regression estimates. *J Clin Epideminol*. 1995:1503-1510.
40. Dempster AP, Laird NM, Rubin DB. Maximum Likelihood from Incomplete Data via the EM Algorithm. *Journal of the Royal Statistical Society Series B (Methodological)*. 1977;39:1-38.
41. Hedeker D, Gibbons RD. Application of random-effects pattern-mixture models for missing data in longitudinal studies. *Psychological Methods*. 1997;2:64-78. doi: 10.1037/1082-989X.2.1.64
42. Miech ED, TM. The Consolidated Framework for Implementation Research: Applying the CFIR Constructs Directly to Qualitative Data. In: *Bi-Annual Meeting: Society for Implementation Research Collaboration*. Seattle, Washington; 2015.
43. Miech ED, TM. Applying the CFIR Constructs Directly to Qualitative Data: The Power of Implementation Science In Action. In: *HSR&D/QUERI National Conference*. Philadelphia, PA; 2015.
44. Damush T, Damschroder L. CFIR Implementation Framework with Application to the VISN11 Stroke Collaborative. In: *VA Cyber-Seminar*. 2010.
45. Walter S, Feinstein A, Wells C. Coding ordinal independent variables in multiple regression analyses. *American Journal of Epidemiology*. 1987;125:319-323.
