# Supplementary material for: Quality Improvement Intervention to Increase Sleep Apnea Diagnostic Testing After Stroke and Transient Ischemic Attack: A Cluster Randomized Trial
Source: JAMA Netw Open. 2025 Nov 14;8(11):e2543385. doi: 10.1001/jamanetworkopen.2025.43385 (PMC12619104; doi:10.1001/jamanetworkopen.2025.43385)
Supplement: Supplement 2. — eAppendix. Imputation Details eTable 1. Exclusions from Cohort: Eligibility for Sleep Apnea Diagnostic Testing eTable 2. Intervention Site Patient Characteristics Over Study Phases eTable 3. Patient Characteristics Across Intervention Sites (N=6) eTable 4. Usual Care Site Patient Characteristics Over Study Data Periods eTable 5. Results of the Sleep Studies at Implementation Sites eTable 6. 30-Day Positive Airway Pressure (PAP) Treatment Rates at Intervention Sites eTable 7. Unadjusted and Adjusted Secondary Outcome Model Results by Study Phase eTable 8. Primary and Secondary Outcomes Adjusted for COVID-19 Burden eTable 9. Primary and Secondary Outcomes Adjusted for Restricted Access to Positive Airway Pressure Devices eTable 10. Primary and Secondary Outcome Model Results Excluding First Implementation Data Period eReferences [file jamanetwopen-e2543385-s002.pdf]

Supplemental Online Content

Bravata DM, Perkins AJ, Myers LJ, et al. Quality improvement intervention to increase sleep apnea diagnostic testing after stroke and transient ischemic attack: a cluster randomized trial. *JAMA Netw Open*. 2025;8(11):e2543385. doi:10.1001/jamanetworkopen.2025.43385

eAppendix. Imputation Details

eTable 1. Exclusions from Cohort: Eligibility for Sleep Apnea Diagnostic Testing

eTable 2. Intervention Site Patient Characteristics Over Study Phases

eTable 3. Patient Characteristics Across Intervention Sites (N=6)

eTable 4. Usual Care Site Patient Characteristics Over Study Data Periods

eTable 5. Results of the Sleep Studies at Implementation Sites

eTable 6. 30-Day Positive Airway Pressure (PAP) Treatment Rates at Intervention Sites

eTable 7. Unadjusted and Adjusted Secondary Outcome Model Results by Study Phase

eTable 8. Primary and Secondary Outcomes Adjusted for COVID-19 Burden

eTable 9. Primary and Secondary Outcomes Adjusted for Restricted Access to Positive Airway Pressure Devices

eTable 10. Primary and Secondary Outcome Model Results Excluding First Implementation Data Period

eReferences

This supplemental material has been provided by the authors to give readers additional information about their work.

© 2025 Bravata DM et al. *JAMA Network Open.*

Supplement A. Imputation Details

The National Institutes of Health Stroke Scale (NIHSS)[1] and 90-Day Care Assessment Needs (CAN) Mortality Score[2] contained 26.0% and 7.5% missing data across all observations, respectively, from the 36 study sites. Multiple-imputation was conducting using fully conditional specification to create 20 imputed datasets.[3] Regression with predictive mean matching (PMM) using the 5 nearest-neighbors was used to impute the NIHSS and 90-Day CAN mortality score so that only plausible values were obtained. Variables included in the imputation model were CHA_2DS_2-VASc score,[4] length of stay, transfer in from an outside hospital, admitted to an intensive care unit, Charlson Comorbidity Index,[5] thrombolytic therapy, history of gait disturbance, hemiplegia, speech deficit, vertigo, dysphagia, congestive heart failure with a B-type natriuretic peptide (BNP) >200 pg/mL, and the Acute Physiology and Chronic Health Evaluation III score (APACHE).[6] This approach assumes data are missing-at-random.

© 2025 Bravata DM et al. *JAMA Network Open.*

eTable 1. Exclusions from Cohort: Eligibility for Sleep Apnea Diagnostic Testing

Exclusion*	Intervention Sites N=2646 (%)	Usual Care Sites N=11,229 (%)
History of obstructive sleep apnea	761 (28.8)	3392 (30.2)
Positive airway pressure in 5-years prior to cerebrovascular event	600 (22.7)	2708 (24.1)
Hypoglossal nerve stimulator	38 (1.4)	147 (1.3)
History of uvulopalatopharyngoplasty	3 (0.1)	87 (0.8)
Oral mandibular device	15 (0.6)	44 (0.4)
Died within seven days of discharge from cerebrovascular event	7 (0.3)	46 (0.4)

*The exclusions were not mutually exclusive.

© 2025 Bravata DM et al. *JAMA Network Open.*

eTable 2. Intervention Site Patient Characteristics Over Study Phases

Patient Characteristics*	Study Phase			P-value
	Baseline	Active Implementation	Sustainability	
Eligible Patients: N	952	650	145	
Age (year): mean (SD)	68.4 (11.0)	68.9 (11.3)	69.3 (11.5)	0.686
median (range)	69 (31-98)	69 (27-98)	69 (39-100)	
Male, n (%)	888 (93.3)	614 (94.5)	132 (91.0)	0.337
Hispanic or Latino Ethnicity, n (%)	46 (4.8)	35 (5.4)	11 (7.6)	0.619
**Race, n (%)**				0.095
Black	403 (42.3)	284 (43.7)	57 (39.3)	
Other	9 (0.9)	20 (3.1)	1 (0.7)	
Unknown	49 (5.2)	35 (5.4)	9 (6.2)	
White	491 (51.6)	311 (47.8)	78 (53.8)	
**Past Medical History, n (%)**				
TIA/Stroke Prior Year	134 (14.1)	114 (17.5)	21 (14.5)	0.282
Hypertension	772 (81.1)	535 (82.3)	116 (80.0)	0.545
Hyperlipidemia	630 (66.2)	442 (68.0)	103 (71.0)	0.533
Diabetes mellitus	419 (44.0)	279 (42.9)	47 (32.4)	0.063
Depression	324 (34.0)	207 (31.8)	48 (33.1)	0.672
COPD	196 (20.6)	124 (19.1)	34 (23.4)	0.558
Congestive Heart Failure	177 (18.6)	125 (19.2)	33 (22.8)	0.575
Myocardial Infarction	74 (7.8)	46 (7.1)	11 (7.6)	0.853
Peripheral Vascular Disease	173 (18.2)	110 (16.9)	22 (15.2)	0.615
Atrial Fibrillation	126 (13.2)	69 (10.6)	19 (13.1)	0.311
Dementia	74 (7.8)	46 (7.1)	11 (7.6)	0.884
History of Weakness	147 (15.4)	138 (21.2)	22 (15.2)	0.035
Charlson Comorbidity Index: median	2 (0-37)	1 (0-19)	1 (0-11)	0.207
**Index Event**				0.910
Stroke	782 (82.1)	531 (81.7)	116 (80.0)	
TIA	170 (17.9)	119 (18.3)	29 (20.0)	
Full Code Status	837 (91.7)	601 (92.5)	132 (91.0)	0.691
Length of Stay (days): median (range)	3.5 (1-126)	4 (1-270)	4 (1-105)	0.132
Admitted to Intensive Care Unit (ICU)	105 (11.0)	63 (9.7)	11 (7.6)	0.530
NIHSS: median (range)	2 (0-37)	2 (0-28)	2 (0-25)	0.543
90-Day CAN Mortality Score: median	70 (0-99)	70 (0-99)	65 (0-99)	0.394
APACHE: median (range)	10 (0-42)	10 (0-42)	9 (0-31)	0.728

*APACHE[6] is a measure of physiological disease severity based on laboratory test data and vital signs; COPD refers to Chronic Obstructive Pulmonary Disease; NIHSS refers to the National Institutes of Health Stroke Scale; SD refers to standard deviation; TIA refers to transient ischemic attack; and the Care Assessment Needs (CAN) score[2] is a measure of mortality risk based on diagnoses and healthcare utilization.

© 2025 Bravata DM et al. *JAMA Network Open.*

eTable 3. Patient Characteristics Across Intervention Sites (N=6)

Patient Characteristics	A	B	C	D	E	F	P-value
Eligible Patients: N	202	236	293	430	375	211	-
Age (year): mean (SD)*	73.3 (10.9)	68.2 (12.2)	68.7 (10.1)	67.9 (10.6)	66.3 (11.1)	70.6 (10.9)	<0.001
median (range)	73 (29-100)	69 (27-96)	68 (34-97)	68 (31-95)	66 (31-99)	71 (39-97)	<0.001
Male sex, n (%)	193 (95.5)	217 (92.0)	274 (93.5)	405 (94.2)	346 (92.3)	199 (94.3)	0.573
Hispanic or Latino Ethnicity, n (%)	3 (1.5)	52 (22.0)	1 (0.3)	31 (7.2)	4 (1.1)	1 (0.5)	<0.001
**Race, n (%)**							<0.001
Black	19 (9.4)	45 (19.1)	158 (53.9)	205 (47.7)	258 (68.8)	59 (28.0)	-
Other[†]	4 (2.0)	4 (1.7)	3 (1.0)	14 (3.3)	3 (0.8)	2 (1.0)	-
Unknown	9 (4.5)	25 (10.6)	12 (4.1)	20 (4.6)	21 (5.6)	6 (2.8)	-
White	170 (84.2)	162 (68.6)	120 (41.0)	191 (44.4)	93 (24.8)	144 (68.2)	-
**Past Medical History, n (%)**							-
TIA/Stroke Prior Year*	23 (11.4)	40 (17.0)	24 (8.2)	76 (17.7)	75 (20.0)	31 (14.7)	<0.001
Hypertension	149 (73.8)	182 (77.1)	256 (87.4)	361 (84.0)	295 (78.7)	180 (85.3)	<0.001
Hyperlipidemia	127 (62.9)	160 (67.8)	186 (63.5)	312 (72.6)	222 (59.2)	168 (79.6)	<0.001
Diabetes mellitus	73 (36.1)	109 (46.2)	130 (44.4)	198 (46.0)	149 (39.7)	86 (40.8)	0.122
Depression	56 (27.7)	87 (36.9)	95 (32.4)	139 (32.3)	131 (34.9)	71 (33.6)	0.426
COPD	40 (19.8)	37 (15.7)	69 (23.6)	89 (20.7)	62 (16.5)	57 (27.0)	0.014
Congestive Heart Failure	36 (17.8)	30 (12.7)	64 (21.8)	100 (23.3)	53 (14.1)	52 (24.6)	<0.001
Myocardial Infarction	17 (8.4)	18 (7.6)	17 (5.8)	24 (5.6)	22 (5.9)	33 (15.6)	<0.001
Peripheral Vascular Disease	43 (21.3)	41 (17.4)	53 (18.1)	74 (17.2)	52 (13.9)	42 (19.9)	0.270
Atrial Fibrillation	44 (21.8)	24 (10.2)	32 (10.9)	43 (10.0)	38 (10.1)	33 (15.6)	<0.001
Dementia	16 (7.9)	18 (7.6)	26 (8.9)	27 (6.3)	21 (5.6)	21 (10.9)	0.203
History of Weakness	41 (20.3)	34 (14.4)	56 (19.1)	71 (16.5)	53 (14.1)	52 (24.6)	0.017
Charlson Comorbidity Index: Median	1 (0-19)	1 (0-11)	1 (0-11)	1 (0-15)	1 (0-13)	1 (0-15)	0.101
**Index Event**							<0.001
Stroke	168 (83.2)	198 (83.9)	259 (88.4)	323 (75.1)	297 (79.2)	184 (87.2)	-
TIA*	34 (16.8)	38 (16.1)	34 (11.6)	107 (24.9)	78 (20.8)	27 (12.8)	-
Full Code Status	161 (79.7)	222 (94.1)	282 (96.2)	403 (93.7)	360 (96.0)	178 (84.4)	<0.001
Length of Stay (days): Median (range)	3.6 (1-136)	2.8 (1-173)	3.4 (1-58)	4.0 (1-270)	3.8 (1-90)	3.0 (1-140)	<0.001
Admitted to Intensive Care Unit (ICU)	14 (6.9)	18 (7.6)	62 (21.2)	28 (6.5)	25 (6.7)	32 (15.2)	<0.001
NIHSS:* Median (range)	2 (0-37)	2 (0-25)	3 (0-33)	2 (0-32)	2 (0-14)	2 (0-24)	0.377
90-Day CAN* Mortality Score: Median	75 (5-99)	65 (0-99)	65 (0-99)	65 (0-99)	70 (0-99)	75 (0-99)	<0.001

© 2025 Bravata DM et al. *JAMA Network Open.*

APACHE:* Median (range)	10 (0-33)	11 (0-33)	11 (0-37)	9 (0-42)	9 (0-42)	10 (0-34)	0.038

*APACHE[6] is a measure of physiological disease severity based on laboratory test data and vital signs; COPD refers to Chronic Obstructive Pulmonary Disease; NIHSS refers to the National Institutes of Health Stroke Scale; SD refers to standard deviation; TIA refers to transient ischemic attack; and the Care Assessment Needs (CAN) score[2] is a measure of mortality risk based on diagnoses and healthcare utilization.

†Other races include: American Indian and Alaska Native, Asian, and Native Hawaiian and Other Pacific Islander.

© 2025 Bravata DM et al. *JAMA Network Open.*

eTable 4. Usual Care Site Patient Characteristics Over Study Data Periods

Patient Characteristics	Data Period				P-value
	1 & 2	3 & 4	5 & 6	7 & 8	
Eligible Patients: N	2168	1875	1756	1655	*
Age (year): mean (SD)	71.4 (11.0)	71.3 (10.6)	72.0 (10.7)	72.8 (10.7)	0.001
median (range)	71 (25-103)	72 (24-101)	72 (22-107)	74 (26-101)	
Male, n (%)	2079 (95.9)	1805 (96.3)	1673 (95.3)	1557 (94.1)	0.015
Hispanic or Latino Ethnicity, n (%)	299 (13.8)	252 (13.4)	227 (12.9)	234 (14.1)	0.748
**Race, n (%)**					0.085
Black	623 (28.7)	574 (30.6)	564 (32.1)	482 (29.1)	
Other	45 (2.1)	44 (2.4)	45 (2.6)	53 (3.2)	
Unknown	84 (3.9)	75 (4.0)	65 (3.7)	75 (4.5)	
White	1416 (65.3)	1182 (63.0)	1082 (61.6)	1045 (63.1)	
**Past Medical History, n (%)**					
TIA/Stroke Prior Year	328 (15.1)	33 (17.8)	284 (16.2)	220 (13.3)	0.005
Hypertension	1760 (81.2)	1543 (82.3)	1378 (78.5)	1315 (79.5)	0.034
Hyperlipidemia	1506 (69.5)	1338 (71.4)	1206 (68.7)	1156 (69.8)	0.357
Diabetes mellitus	929 (42.9)	839 (44.8)	754 (42.9)	710 (42.9)	0.561
Depression	583 (26.9)	519 (27.7)	501 (28.5)	484 (29.2)	0.379
COPD	464 (21.4)	364 (19.4)	336 (19.1)	316 (19.1)	0.292
Congestive Heart Failure	338 (15.6)	318 (17.0)	283 (16.1)	259 (15.7)	0.645
Myocardial Infarction	149 (6.9)	116 (6.2)	125 (7.1)	103 (6.2)	0.634
Peripheral Vascular Disease	397 (18.3)	350 (18.7)	331 (18.9)	270 (16.3)	0.208
Atrial Fibrillation	290 (13.4)	262 (14.0)	266 (15.2)	223 (13.5)	0.367
Dementia	242 (11.2)	161 (8.6)	176 (10.0)	159 (9.6)	0.081
History of Weakness	382 (17.6)	356 (19.0)	335 (19.1)	306 (18.5)	0.632
Charlson Comorbidity Index: median (range)	1 (0-19)	1 (0-16)	1 (0-18)	1 (0-18)	0.142
**Index Event**					0.243
Stroke	1701 (78.5)	1491 (79.5)	1419 (80.8)	1320 (79.8)	
TIA	467 (21.5)	384 (20.5)	337 (19.2)	335 (20.2)	
Full Code Status	1927 (88.9)	1629 (86.9)	1505 (85.7)	1388 (83.9)	<0.001
Length of Stay (days): median (range)	3 (1-135)	3 (1-138)	3 (1-118)	3 (1-159)	0.311
Admitted to Intensive Care Unit (ICU)	220 (10.2)	152 (8.1)	144 (8.2)	128 (7.7)	0.102
NIHSS: median (range)	2 (0-27)	2 (0-35)	2 (0-33)	2 (0-35)	0.178
90-Day Can Mortality Score: median (range)	75 (0-99)	75 (0-99)	75 (0-99)	75 (0-99)	0.078
APACHE: median (range)	10 (0-48)	10 (0-43)	10 (0-50)	10 (0-42)	0.663

*APACHE[6] is a measure of physiological disease severity based on laboratory test data and vital signs; COPD refers to Chronic Obstructive Pulmonary Disease; NIHSS refers to the National Institutes of Health Stroke Scale; SD refers to standard deviation; TIA refers to transient ischemic attack; and the Care Assessment Needs (CAN) score[2] is a measure of mortality risk based on diagnoses and healthcare utilization.

© 2025 Bravata DM et al. *JAMA Network Open.*

eTable 5. Results of the Sleep Studies at Implementation Sites

Sleep Study Result*	N=151 (%)
Obstructive sleep apnea	90 (59.6)
Central sleep apnea	3 (2.0)
Complex sleep apnea	17 (11.3)
No sleep apnea	33 (21.8)
Inconclusive study: full polysomnography recommended	8 (5.3)

*The results of the sleep studies were identified by chart review among N=151 patients at N=6 intervention sites.

© 2025 Bravata DM et al. *JAMA Network Open.*

eTable 6. 30-Day Positive Airway Pressure (PAP) Treatment Rates at Intervention Sites

Intervention Sites	Baseline	Implementation	Sustainability
	Treatment Rate (n/N)<br>(95% Confidence Interval)		
A	2.1% (2/97)<br>95%CI (0.3, 7.3)	3.6% (3/84)<br>95%CI (0.7, 10.1)	0.0% (0/21)<br>95%CI (0.0, 16.1)
B	0.0% (0/95)<br>95%CI (0.0, 3.8)	5.4% (6/111)<br>95%CI (2.0, 11.4)	3.3% (1/30)<br>95%CI (0.1, 17.2)
C	0.0% (0/176)<br>95%CI (0.0, 2.1)	1.2% (1/83)<br>95%CI (0.03, 6.5)	0.0% (0.34)<br>95%CI (0.0, 10.3)
D	0.5% (1/213)<br>95%CI (0.01, 2.6)	3.2% (5/157)<br>95%CI (1.0, 7.3)	0.0% (0/60)<br>95%CI (0.0, 6.0)
E	0.0% (0/228)<br>95%CI (0.0, 1.6)	0.0% (0/147)<br>95%CI (0.0, 2.5)	-
F	0.0% (0/143)<br>95%CI (0.0, 2.6)	4.4% (3/68)<br>95%CI (0.9, 12.4)	-
Overall	0.3% (3/952)<br>95%CI (0.1-0.9)	2.8% (18/650)<br>95%CI (1.7-4.3)	0.7% (1/145)<br>95%CI (0.02, 3.8)

© 2025 Bravata DM et al. *JAMA Network Open.*

eTable 7. Unadjusted and Adjusted Secondary Outcome Model Results by Study Phase

Variables	90 Day Readmission Rate				90 Day Recurrent Vascular Event Rate			
	Unadjusted OR (95% CI)	P-value	Adjusted OR† (95% CI)	P-value	Unadjusted OR (95% CI)	P-value	Adjusted OR† (95% CI)	P-value
**Trial Phase**								
Implementation vs Baseline	0.92 (0.68, 1.24)	0.548	0.89 (0.68, 1.16)	0.383	0.85 (0.56, 1.29)	0.403	0.85 (0.60, 1.22)	0.386
Sustainability vs Baseline	0.62 (0.34, 1.10)	0.091	0.63 (0.38, 1.04)	0.070	0.92 (0.45, 1.89)	0.798	0.96 (0.51, 1.78)	0.887
**Data Period**								
2 vs 1	1.22 (1.02, 1.45)	0.028	1.23 (1.03, 1.47)	0.025	0.95 (0.76, 1.20)	0.687	0.95 (0.76, 1.20)	0.687
3 vs 1	0.96 (0.80, 1.16)	0.690	0.96 (0.79, 1.15)	0.641	0.79 (0.62, 1.01)	0.062	0.79 (0.62, 1.00)	0.055
4 vs 1	0.84 (0.70, 1.01)	0.065	0.84 (0.69, 1.01)	0.062	0.84 (0.66, 1.06)	0.135	0.84 (0.66, 1.06)	0.136
5 vs 1	0.95 (0.78, 1.14)	0.556	0.94 (0.77, 1.14)	0.527	0.73 (0.57, 0.94)	0.014	0.72 (0.56, 0.93)	0.012
6 vs 1	0.93 (0.77, 1.13)	0.477	0.93 (0.76, 1.13)	0.456	0.90 (0.70, 1.15)	0.381	0.89 (0.70, 1.15)	0.377
7 vs 1	0.93 (0.76, 1.13)	0.472	0.93 (0.76, 1.13)	0.455	0.79 (0.62, 1.03)	0.079	0.79 (0.61, 1.03)	0.079
8 vs 1	0.90 (0.73, 1.10)	0.280	0.92 (0.74, 1.12)	0.400	0.76 (0.59, 0.99)	0.045	0.77 (0.59, 1.01)	0.058
Intervention vs Usual Care sites	1.05 (0.85, 1.30)	0.637	1.09 (0.89, 1.32)	0.408	0.94 (0.75, 1.19)	0.610	0.96 (0.77, 1.19)	0.707
**Covariates**								
Neurological symptom severity (NIHSS)*			1.04 (1.03, 1.05)	<0.001			1.04 (1.03, 1.06)	<0.001
90 Day CAN Mortality Score*			1.01 (1.01, 1.02)	<0.001			1.01 (1.01, 1.01)	<0.001
Charlson Comorbidity Index			1.07 (1.05, 1.09)	<0.001			1.06 (1.04, 1.09)	<0.001
History of Congestive Heart Failure			1.29 (1.13, 1.47)	<0.001			1.32 (1.12, 1.56)	0.001
History of Weakness			1.33 (1.18, 1.51)	<0.001				
Full Code Status			1.14 (0.98, 1.32)	0.090				

*NIHSS refers to the National Institutes of Health Stroke Scale. The Care Assessment Needs (CAN) score[2] is a measure of mortality risk based on diagnoses and healthcare utilization.

© 2025 Bravata DM et al. *JAMA Network Open.*

†Multiple-imputation results.

© 2025 Bravata DM et al. *JAMA Network Open.*

eTable 8. Primary and Secondary Outcomes Adjusted for COVID-19 Burden

Variables	30-Day Diagnostic Testing Rate		90-Day Readmission Rate		90-Day Recurrent Event Rate	
	Adjusted OR (95% CI)	P-value	Adjusted OR (95% CI)	P-value	Adjusted OR (95% CI)	P-value
**Trial Phase**						
Implementation vs Baseline	17.03 (8.66, 33.49)	<0.001	0.93 (0.69, 1.26)	0.608	0.85 (0.56, 1.29)	0.400
Sustainability vs Baseline	3.52 (1.37, 9.04)	0.015	0.62 (0.35, 1.11)	0.096	0.92 (0.45, 1.89)	0.794
**Data Period**						
2 vs 1	0.87 (0.47, 1.59)	0.649	1.21 (1.01, 1.44)	0.034	0.96 (0.76, 1.20)	0.692
3 vs 1	0.70 (0.36, 1.35)	0.287	0.94 (0.77, 1.14)	0.516	0.80 (0.62, 1.03)	0.084
4 vs 1	0.68 (0.38, 1.22)	0.196	0.83 (0.69, 1.00)	0.048	0.84 (0.66, 1.07)	0.151
5 vs 1	0.60 (0.33, 1.12)	0.108	0.91 (0.74, 1.12)	0.370	0.73 (0.56, 0.97)	0.028
6 vs 1	0.78 (0.43, 1.39)	0.390	0.90 (0.73, 1.11)	0.324	0.90 (0.69, 1.18)	0.457
7 vs 1	1.46 (0.85, 2.52)	0.168	0.91 (0.75, 1.12)	0.369	0.80 (0.61, 1.04)	0.094
8 vs 1	0.98 (0.53, 1.79)	0.940	0.88 (0.72, 1.08)	0.218	0.77 (0.59, 1.00)	0.053
Intervention vs Usual Care sites	1.70 (0.84, 3.43)	0.137	1.05 (0.85, 1.30)	0.638	0.94 (0.75, 1.19)	0.611
COVID-19 Rate*	1.01 (1.00, 1.01)	0.113	1.00 (1.00, 1.00)	0.382	1.00 (1.00, 1.00)	0.858

*To examine the potential effect of the COVID-19 pandemic on the trial results, we included a time-varying measure of COVID-19 burden calculated as the total number of COVID-19 patients per data period per site.

© 2025 Bravata DM et al. *JAMA Network Open.*

eTable 9. Primary and Secondary Outcomes Adjusted for Restricted Access to Positive Airway Pressure Devices

Variables	30-Day Diagnostic Testing Rate		90-Day Readmission Rate		90-Day Recurrent Event Rate	
	OR (95% CI)	P-value	OR (95% CI)	P-value	OR (95% CI)	P-value
**Trial Phase**						
Implementation vs Baseline	16.38 (8.37, 32.06)	<0.001	0.92 (0.68, 1.24)	0.540	0.85 (0.56, 1.29)	0.397
Sustainability vs Baseline	3.37 (1.31, 8.64)	0.018	0.61 (0.34, 1.10)	0.090	0.92 (0.45, 1.90)	0.800
**Data Period**						
2 vs 1	0.92 (0.50, 1.69)	0.786	1.22 (1.02, 1.45)	0.028	0.95 (0.76, 1.20)	0.687
3 vs 1	0.82 (0.43, 1.54)	0.529	0.96 (0.80, 1.16)	0.690	0.79 (0.62, 1.01)	0.062
4 vs 1	0.68 (0.37, 1.26)	0.219	0.87 (0.71, 1.06)	0.167	0.85 (0.66, 1.09)	0.202
5 vs 1	1.13 (0.48, 2.65)	0.775	1.08 (0.75, 1.55)	0.666	0.55 (0.35, 0.88)	0.013
6 vs 1	0.93 (0.50, 1.72)	0.805	1.00 (0.79, 1.26)	0.984	0.84 (0.62, 1.13)	0.250
7 vs 1	1.64 (0.96, 2.80)	0.067	0.93 (0.77, 1.13)	0.474	0.80 (0.62, 1.03)	0.079
8 vs 1	1.09 (0.60, 1.98)	0.779	0.90 (0.73, 1.10)	0.282	0.76 (0.59, 0.99)	0.045
Intervention vs Usual Care sites	1.71 (0.85, 3.45)	0.130	1.05 (0.85, 1.30)	0.637	0.94 (0.75, 1.19)	0.604
Restricted PAP Access Status*						
Red vs Green	0.62 (0.32, 1.23)	0.170	0.87 (0.63, 1.21)	0.404	1.33 (0.89, 2.00)	0.165
Yellow vs Green	1.34 (0.82, 2.17)	0.238	0.91 (0.73, 1.13)	0.390	0.96 (0.72, 1.28)	0.797

*During the Philips Respironics Continuous Positive Airway Pressure (CPAP) device recall, the VA entered a period of restricted access to PAP devices: the red period was the most restrictive, yellow being intermediate (stroke patients with OSA could receive PAP devices), and green being a period where devices were readily available.

© 2025 Bravata DM et al. *JAMA Network Open.*

eTable 10. Primary and Secondary Outcome Model Results Excluding First Implementation Data Period

Variables	30-Day Diagnostic Testing Rate		90-Day Readmission Rate		90-Day Recurrent Event Rate	
	OR (95% CI)	P-value	OR (95% CI)	P-value	OR (95% CI)	P-value
**Trial Period**						
Implementation vs Baseline	21.37 (10.34, 44.19)	<0.001	0.87 (0.61, 1.23)	0.377	0.65 (0.39, 1.10)	0.094
Sustainability vs Baseline	3.18 (1.23, 8.23)	0.023	0.60 (0.33, 1.07)	0.076	0.91 (0.44, 1.88)	0.770
**7 Month Data Period**						
2 vs 1	0.92 (0.50, 1.69)	0.789	1.22 (1.02, 1.45)	0.029	0.95 (0.76, 1.20)	0.687
3 vs 1	0.82 (0.43, 1.55)	0.535	0.96 (0.80, 1.16)	0.684	0.79 (0.62, 1.01)	0.062
4 vs 1	1.14 (0.63, 2.07)	0.656	0.84 (0.69, 1.01)	0.059	0.82 (0.64, 1.03)	0.092
5 vs 1	0.44 (0.22, 0.87)	0.018	0.96 (0.79, 1.16)	0.637	0.73 (0.56, 0.94)	0.016
6 vs 1	0.96 (0.54, 1.73)	0.899	0.92 (0.75, 1.12)	0.397	0.89 (0.69, 1.14)	0.360
7 vs 1	1.50 (0.86, 2.60)	0.152	0.94 (0.77, 1.15)	0.541	0.82 (0.64, 1.06)	0.138
8 vs 1	1.02 (0.55, 1.88)	0.951	0.90 (0.73, 1.10)	0.296	0.78 (0.60, 1.01)	0.059
Intervention vs Usual Care sites	1.67 (0.81, 3.46)	0.162	1.05 (0.85, 1.30)	0.634	0.95 (0.75, 1.20)	0.656

© 2025 Bravata DM et al. *JAMA Network Open.*

eREFERENCES

1. Kogan E, Twyman K, Heap J, Milentijevic D, Lin JH, Alberts M. Assessing stroke severity using electronic health record data: a machine learning approach. *BMC Med Inform Decis Mak*. Jan 8 2020;20(1):8. doi:10.1186/s12911-019-1010-x
2. Wong ES, Yoon J, Piegari RI, Rosland AM, Fihn SD, Chang ET. Identifying Latent Subgroups of High-Risk Patients Using Risk Score Trajectories. *J Gen Intern Med*. Dec 2018;33(12):2120-2126. doi:10.1007/s11606-018-4653-x
3. van Buuren S. Multiple imputation of discrete and continuous data by fully conditional specification. *Stat Methods Med Res*. Jun 2007;16(3):219-42. doi:10.1177/0962280206074463
4. van Doorn S, Debray TPA, Kaasenbrood F, et al. Predictive performance of the CHA2DS2-VASc rule in atrial fibrillation: a systematic review and meta-analysis. *J Thromb Haemost*. Jun 2017;15(6):1065-1077. doi:10.1111/jth.13690
5. Charlson M, Pompei P, Ales K, MacKenzie C. A new method of classifying prognostic comorbidity in longitudinal studies: development and validation. *Journal of Chronic Diseases*. 1987;40:373-383.
6. Knaus W, Wagner D, Draper E, et al. The APACHE III prognostic system. Risk prediction of hospital mortality for critically ill hospitalized adults. *Chest*. 1991;100:1619-1636.

© 2025 Bravata DM et al. *JAMA Network Open.*
