# Supplementary material for: Quality Improvement Intervention to Increase Sleep Apnea Diagnostic Testing After Stroke and Transient Ischemic Attack: A Cluster Randomized Trial
Source: JAMA Netw Open. 2025 Nov 14;8(11):e2543385. doi: 10.1001/jamanetworkopen.2025.43385 (PMC12619104; doi:10.1001/jamanetworkopen.2025.43385)
Supplement: Supplement 3. — Nonauthor Collaborators [file jamanetwopen-e2543385-s003.pdf]

Supplemental Online Content: Nonauthor Collaborators
*First name, last name, and suffix (if applicable) are required and will appear in PubMed.

*Group Name(s): ASAP INVESTIGATORS GROUP							
***First Name and Middle Initial(s)**	***Last Name**	***Suffix (eg, Jr, III)**	Academic Degrees	Institution	Location (city, state/province, country)	Role or Contribution, eg, chair, principal investigator	Group (if more than 1 Group listed in the byline) and/or Subgroup (eg, Steering Committee)
Joseph T.	Daley		PhD, MD	Department of Neurology, Birmingham VA Medical Center<br><br>Department of Neurology, University of Alabama at Birmingham School of Medicine	Birmingham, AL	Local Site Investigator; Birmingham	ASAP Investigators Group
Barry G.	Fields		MD	Division of Pulmonary, Allergy, Critical Care and Sleep Medicine, Emory University School of Medicine<br><br>Department of Medicine, Sleep Medicine Center, Atlanta VA Medical Center	Atlanta, GA	Local Site Investigator; Atlanta	ASAP Investigators Group
Lisa D.	Hermann		MD	Department of Neurology, VA Tennessee Valley Health Care System<br><br>Department of Neurology, Vanderbilt University	Nashville, TN	Local Site Investigator; Nashville	ASAP Investigators Group
Ken M.	Kunisaki		MD	Section of Pulmonary, Allergy, Critical Care and Sleep, Minneapolis VA Health Care System<br><br>Division of Pulmonary, Allergy, Critical Care and Sleep, University of Minnesota	Minneapolis, MN	Local Site Investigator; Minneapolis	ASAP Investigators Group

Supplemental Online Content: Nonauthor Collaborators
*First name, last name, and suffix (if applicable) are required and will appear in PubMed.

***First Name and Middle Initial(s)**	***Last Name**	***Suffix (eg, Jr, III)**	Academic Degrees	Institution	Location (city, state/province, country)	Role or Contribution, eg, chair, principal investigator	Group (if more than 1 Group listed in the byline) and/or Subgroup (eg, Steering Committee)
Santiago	Palacio		MD	South Texas Veterans Health Care System, San Antonio, TX, USA	San Antonio, TX	Local Site Investigator; San Antonio	ASAP Investigators Group
