# Supplementary material for: Quality Improvement Intervention to Increase Sleep Apnea Diagnostic Testing After Stroke and Transient Ischemic Attack: A Cluster Randomized Trial
Source: JAMA Netw Open. 2025 Nov 14;8(11):e2543385. doi: 10.1001/jamanetworkopen.2025.43385 (PMC12619104; doi:10.1001/jamanetworkopen.2025.43385)
Supplement: Supplement 4. — Data Sharing Statement [file jamanetwopen-e2543385-s004.pdf]

Data Sharing Statement

Bravata. Quality Improvement Intervention to Increase Sleep Apnea Diagnostic Testing After Stroke and Transient Ischemic Attack. *JAMA Netw Open*. Published November 14, 2025. doi:10.1001/jamanetworkopen.2025.43385

Data
Additional Information: ClinicalTrials.gov NCT04322162
Data available: No

Additional Information
Explanation for why data not available: The data that support the findings of this study must remain on Department of Veterans Affairs servers. Please contact the corresponding author if you are interested in working with these data.
